# Defined YNB-free mineral medium improves reproducibility and enables high-titer production in *Yarrowia lipolytica*

**DOI:** 10.1186/s12934-026-02939-6

**Published:** 2026-02-03

**Authors:** Demian Dietrich, Hang Qi, Sofija Jovanovic Gasovic, Michael Kohlstedt, Christoph Wittmann

**Affiliations:** Institute of Systems Biotechnology, Saarland University, Saarbrücken, Germany

**Keywords:** Yarrowia lipolytica, Medium engineering, Yeast nitrogen base, Design of experiments, Flaviolin reporter, acetyl-CoA and malonyl-CoA metabolism, Omega-3 fatty acids, Zinc and iron homeostasis

## Abstract

**Background:**

*Yarrowia lipolytica* is an emerging host for producing acetyl-CoA– and malonyl-CoA–derived chemicals. However, most processes rely on yeast nitrogen base (YNB), a historical formulation with poorly controlled trace metal content. This variability impairs metabolic performance, limits reproducibility, and complicates process transfer.

**Results:**

Commercial YNB batches differed markedly, causing 1.5–2-fold variation in growth and docosahexaenoic acid (DHA) production. We developed a malonyl-CoA–responsive flaviolin reporter strain and combined it with a structured Design of Experiments (DoE) workflow to systematically re-engineer YNB mineral composition. Dissection of all 20 YNB components revealed that vitamins are dispensable under the tested conditions, whereas a small subset of salts and trace elements - particularly ZnSO_4_, FeCl_3_, KH_2_PO_4_, MgSO_4_, CaCl_2_, and CuSO_4_ - dominantly shape precursor availability and product formation. One-factor-at-a-time (OFAT), factorial, steepest ascent, and central composite designs converged in an optimized synthetic mineral medium assembled entirely from individual salts and trace metals. This formulation increased flaviolin titers to 1.41 ± 0.08 g L^-1^, a more-than threefold improvement over commercial YNB, while ensuring high reproducibility. Key mineral interventions also translated to complex pathways: omission of ZnSO_4_ increased PUFA titers by 7.6-fold (docosapentaenoic acid, DPA) and 58-fold (eicosapentaenoic acid, EPA) and enhanced DHA formation in independent production strains. The defined formulation substantially reduces cost and eliminates batch-to-batch variability inherent to commercial YNB powders.

**Conclusions:**

Our results establish mineral balancing as a major yet underused lever for improving acetyl-CoA– and malonyl-CoA–derived production in *Y. lipolytica* and demonstrate a generalizable, model-guided workflow for creating simplified, reproducible, and cost-efficient synthetic media for non-conventional yeast cell factories.

**Supplementary Information:**

The online version contains supplementary material available at 10.1186/s12934-026-02939-6.

## Background

Yeasts are central workhorses in industrial biotechnology, supporting the production of fuels, bioplastics, lipids, enzymes, pharmaceuticals, and nutraceuticals. Among them, *Yarrowia lipolytica* is a particularly versatile host [1], valued for its metabolic flexibility, robustness, and ability to generate a wide range of acetyl-CoA- and malonyl-CoA–derived products [2]. Many academic and industrial processes with *Y. lipolytica* are carried out in chemically defined media, most commonly using yeast nitrogen base (YNB) as the basal mineral formulation (Table 1). Since its introduction nearly 80 years ago [3], YNB has become the standard mineral medium because it is convenient to use and broadly compatible with different yeast species.

**Table 1:** YNB-based media used for production in industrially relevant yeasts

Host organism	Target product	Role of YNB-based defined medium	Reference
*Y. lipolytica*	Lipids and biofuels	Standard defined production medium	[6]
*Y. lipolytica*	Fatty acid-derived oleochemicals and biofuels	YNB-based medium for pathway optimization and production	[7]
*Y. lipolytica*	Long-chain polyunsaturated fatty acids (DHA / EPA)	Minimal YNB-glycerol (YNBG) and related YNB-based production medium	[9, 38]
*S. cerevisiae*	Volatile aroma compounds	YNB-based defined medium	[39]
*P. pastoris*	Heterologous membrane protein (human A2A receptor)	Standard defined bioreactor medium (BMGY/BMMY)	[40]
*K. marxianus*	Ethanol and organic acids	Defined YNB-glucose medium	[41]

The media include minimal synthetic media formulated directly from YNB as the mineral backbone, as well as widely used production media (e.g., YNBG, BMGY/BMMY) that incorporate YNB as a defined mineral component

Despite its widespread use, YNB presents two important limitations for modern bioprocess development. First, its formulation has changed little since its original design, which focused on supporting general yeast growth rather than maximizing product formation [4]. Second, commercial YNB powders show notable variation in their trace metal content across suppliers and even across batches from the same manufacturer, leading to measurable differences in growth and metabolic performance [5]. The practical relevance of this issue became evident in our own work. During ongoing strain and process development, switching to a newly purchased batch of commercial YNB caused a pronounced decline in growth and product formation, and previously obtained production data could no longer be reproduced. Only through systematic troubleshooting did batch-to-batch variation in YNB composition emerge as the underlying cause. This experience highlighted that even subtle, undocumented changes in YNB composition can critically affect engineered *Y. lipolytica* performance and motivated a systematic evaluation of individual medium components.

The impact of such variability is amplified by the fact that YNB-based defined media are widely used not only for screening but also as production media in *Y. lipolytica*, supporting production of lipids and biofuels [6], hydrocarbons [7], pharma proteins [8], and long-chain (LC) polyunsaturated fatty acids [9]. These LC-PUFAs, including EPA and DHA, represent high-value ingredients for aquafeed, “superfoods”, and pharmaceutical applications [10]. Consequently, batch-to-batch variation in YNB composition has the potential to compromise reproducibility and comparability across laboratories and process stages, complicating strain engineering efforts and undermining confidence in reported production metrics - an increasing concern in both research and regulated industrial settings [11]. Systematic analysis of individual YNB components, and medium redesign tailored to *Y. lipolytica* physiology, has therefore become increasingly important.

In our previous work engineering *Y. lipolytica* for omega-3 fatty acid production, we also found that the overall concentration of YNB strongly affected product formation. In that study, reducing the YNB level significantly improved DHA titers in the producer strain Af4 [9]. This observation suggested that not only the composition but also the amount of YNB can shape metabolic performance, further motivating a systematic evaluation of YNB components.

Yet, while many production targets can in principle be screened in a high-throughput manner based on product accumulation, medium optimization remains challenging when intracellular precursor availability—rather than the final product itself—is the key performance determinant. This is particularly true for acetyl-CoA– and malonyl-CoA–derived pathways, where precursor supply and cofactor balance often limit productivity long before end-product accumulation becomes measurable [12].

To address these gaps, we combined a simple product-linked reporter system with a structured medium-engineering workflow. By integrating the *rppA* gene from *Streptomyces griseus*, which encodes a type III polyketide synthase (PKS), we generated *Y. lipolytica* strains that produce flaviolin—a red pigment whose intensity directly reflects acetyl-CoA/malonyl-CoA flux [13]. Beyond serving as an intracellular sensor for acetyl-CoA/malonyl-CoA availability, flaviolin also functions as a representative polyketide product, making it a suitable model for evaluating how medium composition directly affects PKS-driven chemical production. Using this reporter in a micro-bioreactor platform, we systematically dissected the contributions of all 20 YNB components. Through a stepwise sequence of experiments - including compound-class screening, one-factor-at-a-time tests, two-level factorial designs, steepest ascent analysis, and response surface modeling (RSM) following established DoE methodologies [14] - we identified which components were essential, which were beneficial, and which inhibited production.

This approach enabled us to replace standard commercial YNB premixes with a simplified, YNB-free synthetic mineral medium, in which the mineral composition is reconstructed from individual salts and trace metals and prepared entirely without costly YNB powders or vitamins. Beyond improving flaviolin titers, we tested the broader robustness of this optimized medium in *Y. lipolytica* strains engineered for omega-3 fatty acid biosynthesis via myxobacterial polyketide-like PUFA synthases—pathways that are complex, cofactor-demanding, and highly sensitive to medium composition [9, 12, 15]. These strains provide a stringent test case for assessing the robustness of medium design.

Our findings provide a practical alternative to commercial YNB and demonstrate a generalizable workflow for medium engineering in non-conventional yeasts. While the optimized mineral composition described here is not intended as a universal medium for all *Y. lipolytica* products, it provides a robust and reproducible baseline specifically tailored to acetyl-CoA– and malonyl-CoA–dependent biosynthetic pathways.

## Results

### Commercial YNB variability causes major shifts in growth and product formation

Commercial YNB is widely used as the mineral base for *Y. lipolytica* fermentations, yet earlier work has shown that its composition—especially trace metal content—can vary considerably across manufacturers and batches [5]. Because such variability can influence cellular metabolism, we examined whether different commercial YNB formulations also affect the performance of engineered *Y. lipolytica* strains producing omega-3 fatty acids.

**Fig. 1:**
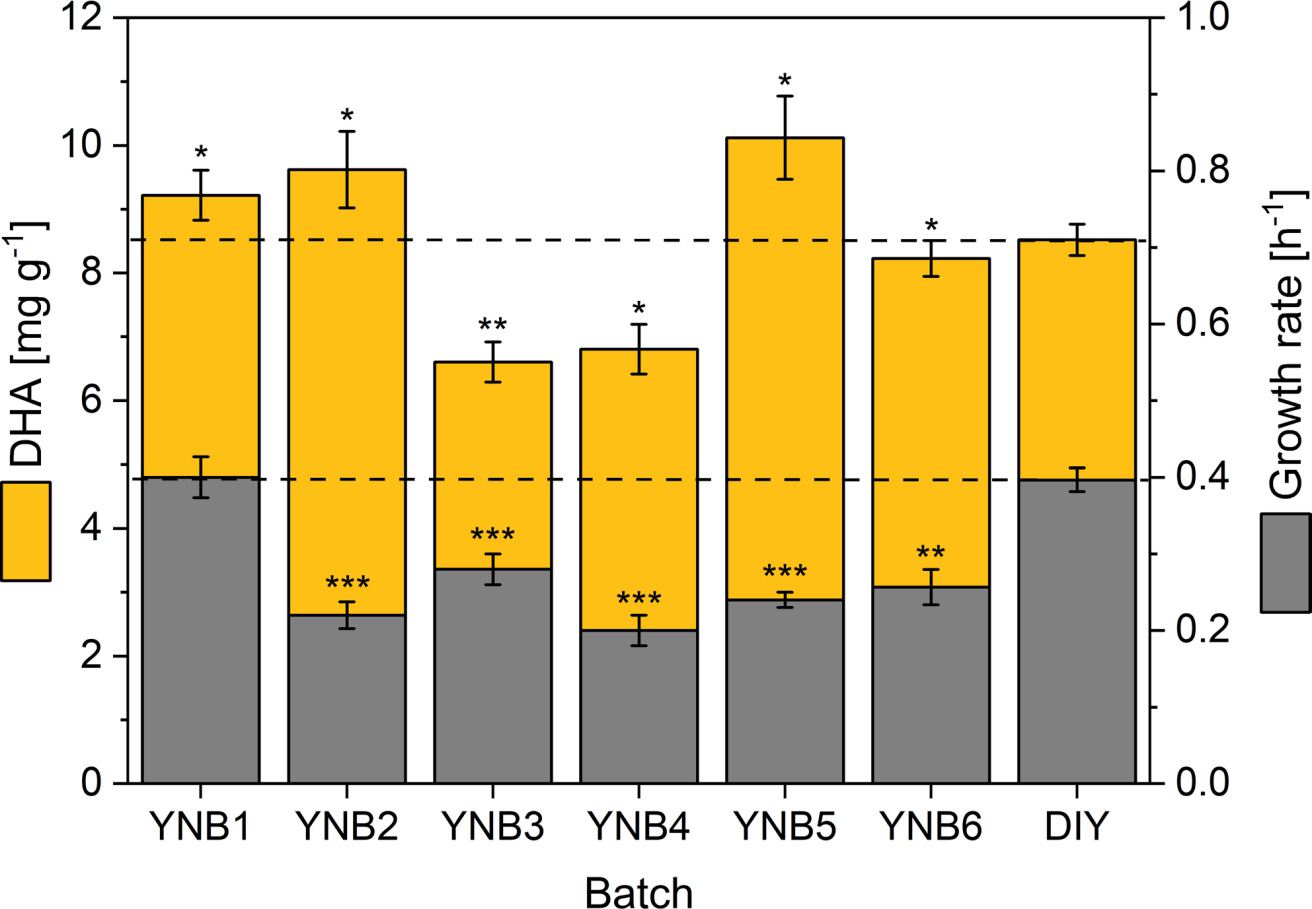
Variability of commercial YNB batches affects growth and DHA production in *Y. lipolytica*. Specific growth rates (left) and DHA production (right) of the DHA-producing strain *Y. lipolytica* Af4 cultivated in shake flasks with different commercial YNB batches and a defined lab-made YNB (DIY). YNB1–YNB6 represent recently ordered batches from three manufacturers: YNB1–YNB3, Manufacturer 1 (Lots 1–3); YNB4–YNB5, Manufacturer 2 (Lots 1–2); YNB6, Manufacturer 3 (Lot 1). DIY YNB was prepared from 20 individually controlled stock solutions for each YNB component. Growth rates were determined from inoculation until an OD_600_ of 8 was reached. DHA end concentrations were quantified by GC–MS after 7 days. Data represent means ± standard deviation (SD) of biological triplicates. Statistical significance was assessed by two-sided *t*-tests comparing the indicated condition to the respective reference (1×) concentration and is denoted as *p* < 0.05 (*),* p* < 0.01 (**), and *p* < 0.001 (***). CDW, cell dry weight; DHA, docosahexaenoic acid (C22:6); YNB, yeast nitrogen base

To test this, we compared six YNB batches from three suppliers using the DHA-producing strain Af4 [15]. For each batch, specific growth rates and flaviolin end-point titers were measured under identical cultivation conditions. As shown in Fig. 1, both parameters varied markedly across the commercial YNBs. The DHA titer differed by up to 1.5-fold, while growth rates fluctuated even more strongly, up to 2-fold. Importantly, these responses did not correlate: some batches supported rapid growth but low DHA formation, whereas others showed the opposite pattern. This demonstrated that YNB components influence growth and product formation through distinct metabolic pathways rather than through a simple growth-dependent effect.

Such inconsistent performance presents a challenge for quantitative process development, where stable nutrient composition is essential for obtaining reproducible production metrics. To overcome this issue, we prepared a lab-made YNB formulation assembled from individual salts, trace elements, and vitamins at defined concentrations (DIY YNB). Three concentrated stock solutions representing the main component classes - vitamins (100×), trace elements (100×), and salts (10×) - were prepared, along with individual 100× stocks for each component when non-standard levels were required (Additional File 1, Table S1). This synthetic YNB minimized variability and ensured precise control over mineral composition, yielding consistent growth and DHA production across independently prepared batches and providing a robust baseline for all subsequent medium-optimization experiments. In subsequent optimization steps, we used this component-resolved system to derive a defined YNB-free mineral medium, formulated from individually dosed salts and trace metals without any commercial YNB premix or vitamin mixture. Each step in this workflow was designed to progressively reduce factor space, quantify dominant effects, and refine concentrations toward an experimentally validated optimum (Additional File 1; Table S2, Table S3).

### A flaviolin reporter enables rapid, product-linked medium evaluation

**Fig. 2:**
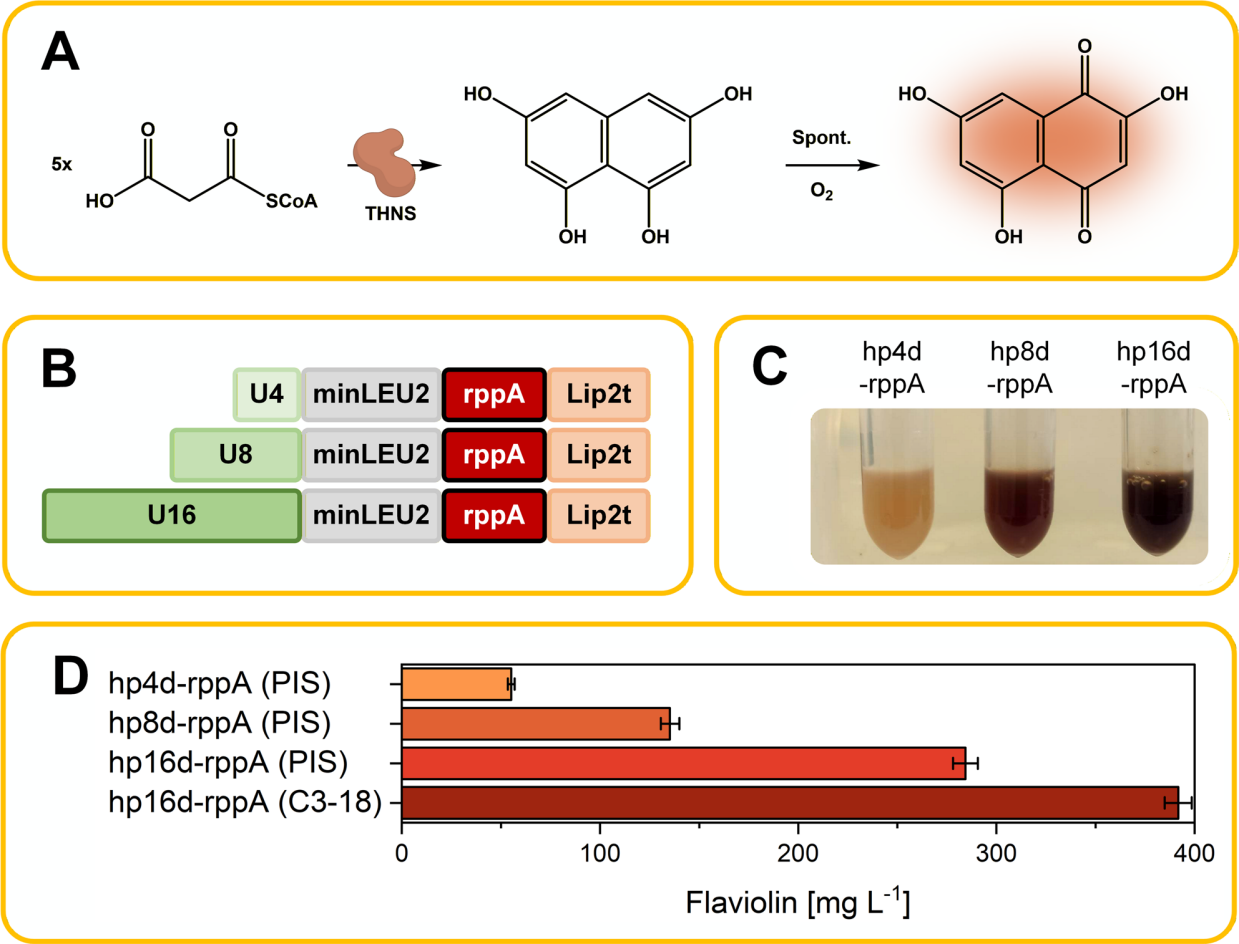
Flaviolin-based reporter system for medium screening in *Y. lipolytica*.** A** Schematic of flaviolin biosynthesis from malonyl-CoA. The type III polyketide synthase 1,3,6,8-tetrahydroxynaphthalene (THNS) synthase (gene *rppA* from *Streptomyces griseus* NBRC 13350) converts five molecules of malonyl-CoA into 1,3,6,8-tetrahydroxynaphthalene (THN), which spontaneously oxidizes to flaviolin.** B** Design of the *rppA* expression cassettes. Each linear integration fragment contains the *minLEU2* core promoter, *rppA* coding sequence, and *Lip2* terminator. Promoter strength was tuned by placing 4, 8, or 16 tandem repeats of the upstream activating sequence UAS1B upstream of *minLEU2*, yielding hp4d–*rppA*, hp8d–*rppA*, and hp16d–*rppA*, respectively. All clusters were integrated by homologous recombination (HR) into the integration site (PIS).** C** Photographs of culture supernatants after 144 h cultivation of *Y. lipolytica* hp4d–*rppA*, hp8d–*rppA*, and hp16d–*rppA* strains in defined medium, illustrating progressively stronger red pigmentation with increasing UAS1B copy number.** D** Flaviolin titers of the three PIS-based producer strains after 144 h, quantified spectrophotometrically at 520 nm. For comparison, the titer of strain C3-18 - carrying the hp16d–*rppA* cassette integrated by random non-homologous end joining (NHEJ) and selected for highest pigment formation - is shown alongside the PIS-based strains. C3-18 was used as the reporter strain for all subsequent medium-optimization experiments due to its superior production performance. Data represent means ± SD of biological triplicates

To enable rapid and quantitative evaluation of medium components, we established a colorimetric reporter system based on flaviolin production. For this purpose, the *rppA* gene from *Streptomyces griseus*, which encodes a type III polyketide synthase that converts malonyl-CoA into the red pigment flaviolin, was introduced into *Y. lipolytica*. Because flaviolin formation depends directly on intracellular acetyl-CoA and malonyl-CoA supply [16], its visible red coloration provides an immediate and high-throughput proxy for precursor flux (Fig. 2A). Importantly, the flaviolin reporter does not represent general cellular fitness but specifically reports on intracellular acetyl-CoA and malonyl-CoA availability. Consequently, the optimization strategy and resulting medium composition are expected to be most relevant for pathways that share similar precursor and cofactor requirements, rather than for all possible products in *Y. lipolytica*.

To obtain a strong and reliable pigment signal for medium screening, we first constructed three *rppA* expression cassettes driven by hybrid promoters containing 4, 8, or 16 copies of the UAS1B enhancer (hp4d, hp8d, and hp16d) and integrated each cassette into the YALI0_C05907g locus, previously identified as preferred integration site (PIS) for polyketide-synthase-driven bacterial gene clusters in *Y. lipolytica* [9]. This design allowed a clean comparison of promoter architectures. As expected, increasing the number of UAS1B elements led to progressively higher flaviolin formation (Fig. 2C–D), with the hp16d promoter yielding the highest and most robust pigment levels. Across biological triplicates in micro-bioreactor cultivations, all PIS-based reporter strains showed low replicate-to-replicate variability, with coefficients of variation (CVs) of 2.2% for hp16d–rppA, 3.5% for hp8d–rppA, and 2.5% for hp4d–rppA. Based on this defined comparison, hp16d–*rppA* was selected as the reporter cassette for subsequent medium engineering.

To maximize the dynamic range and sensitivity of the reporter for Design of Experiments (DoE) studies, we next generated a library of hp16d–*rppA* strains in which the expression cassette was integrated randomly into the genome via non-homologous end joining. In this context, copy number and genomic insertion site were intentionally not controlled, as the goal was not to compare absolute expression levels but to identify a high-output, phenotypically robust reporter strain with maximal signal-to-noise for medium screening. From this library, strain hp16d–*rppA* C3-18 was selected because it consistently produced the high flaviolin titers with low well-to-well variability in micro-bioreactor cultivation (Fig. 2D). Importantly, despite its high output, flaviolin production in C3-18 varied strongly across medium conditions, indicating that the reporter signal was not saturated and retained sufficient dynamic range to resolve mineral-dependent effects. This high-output strain therefore served exclusively as a sensitive sensor for medium effects in all DoE-based optimization experiments, where a large signal window is crucial for robustly identifying influential factors. Like the PIS-based reporter strains, the randomly integrated reporter strain hp16d–rppA C3-18 exhibited low variability in microbioreactor cultivation (CV = 1.7%), underscoring its robustness as a high-signal sensor strain.

By linking pigment formation directly to intracellular precursor availability, this reporter system enabled fast, scalable, and visually quantifiable assessment of medium compositions, forming the basis of the optimization workflow described in the following sections. Importantly, key medium trends identified with hp16d–*rppA* C3-18 were later confirmed in the original PIS-based hp16d-*rppA* strain and in independent LC-PUFA production strains (see below), demonstrating that the optimized formulation is not specific to a single integration site or strain background.

### Component-group screening reveals vitamins are dispensable and metal salts drive productivity

With the flaviolin reporter strain established, we first examined how the major component groups of YNB - vitamins, salts, and trace elements (Table S1, Additional File 1) - affect flaviolin production. Grouping components at this stage allowed us to detect broad functional trends before moving to more detailed single-component testing, ensuring an efficient and structured optimization workflow. To do this, we performed a full factorial screening experiment in which each group was tested at reduced (0.5×), standard (1×), and elevated (2×) concentrations, resulting in 27 medium combinations evaluated in the micro-bioreactor system.

**Fig. 3:**
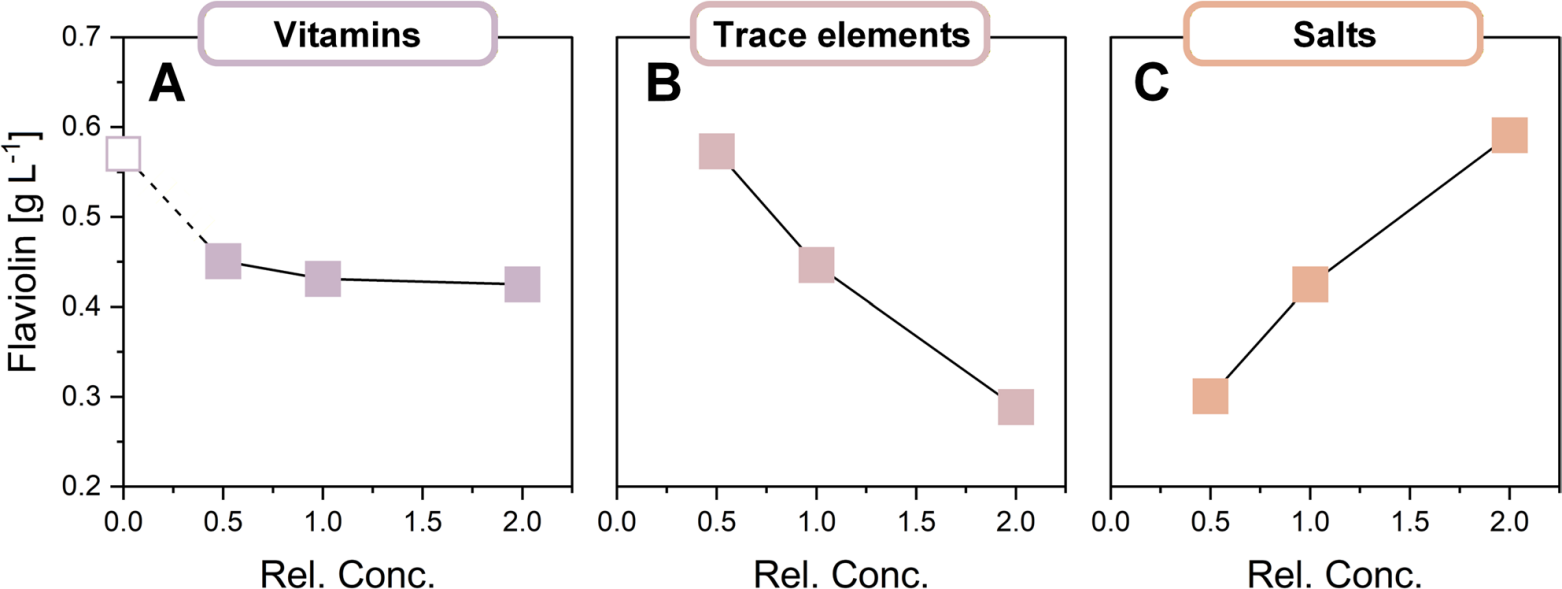
Group-wise screening of YNB vitamins, trace elements, and salts for effects on flaviolin production. Effects of the three major YNB component classes—vitamins (**A**), trace elements (**B**), and salts (**C**)—on flaviolin production by the reporter strain *Y. lipolytica* C3-18. Concentrations of each class were set to 0.5×, 1×, or 2× of the standard YNB level in a full factorial design (3^3^; 27 combinations), yielding 54 randomized runs (each condition in duplicate). An additional condition without vitamins (open symbol in A) was included. Cultivations were performed in a micro-bioreactor system for 144 h, and flaviolin titers were quantified in the supernatant. The full statistical evaluation of the factorial design, including the experimental matrix and ANOVA-based significance analysis, is provided in Additional File 2

The results revealed clear differences in how the component groups influenced flaviolin production (Fig. 3). Vitamin levels had only a minor effect, with slightly higher flaviolin titers observed at reduced concentrations. This modest positive response prompted an additional experiment in which vitamins were omitted entirely. Strikingly, removing all vitamins increased flaviolin production from 0.43 g L^− 1^ to 0.57 g L^− 1^, confirming that vitamins are not required under the tested conditions and may even inhibit production. Because vitamins make up nearly half of all YNB components, their omission substantially simplified the subsequent optimization workflow. In contrast, salts and trace elements showed strong and opposing effects. Higher salt concentrations generally boosted flaviolin production, whereas higher trace element concentrations consistently decreased it. The two groups also interacted noticeably. Factorial ANOVA, testing the vitamin × salt (*p* = 0.94), vitamin × trace element (*p* = 0.51), and salt × trace element (*p* = 8 × 10^− 5^) interaction terms, also identified a significant interaction between salts and trace elements. This indicated that their combined influence was not simply additive and underscored the need to examine individual components within each group.

Whereas flaviolin production changed markedly across conditions, no obvious growth defects were observed during micro-bioreactor cultivation, indicating that medium composition primarily affected pathway flux rather than biomass formation. Because the reporter was designed to probe intracellular acetyl-CoA/malonyl-CoA flux rather than growth-coupled productivity, biomass-normalized metrics were not used as a primary optimization criterion in this study.

Overall, this component-group screen showed that (i) vitamins can be omitted without compromising performance, (ii) salts tend to support flaviolin formation, and (iii) trace elements include compounds with substantial inhibitory effects. Across the tested medium conditions, changes in flaviolin production were generally much larger than differences in growth. While biomass formation was not systematically quantified for all DoE conditions, routine cultivation observations and endpoint measurements indicated that growth differences between media were modest compared to the several-fold changes observed in product titers. This supports the conclusion that mineral composition primarily modulates pathway productivity rather than biomass accumulation under the tested conditions. Because this analysis identified strong functional differences at the group level but could not resolve the contribution of individual components, we next examined each salt and trace element separately. This step reduced the factor space and ensured that subsequent multifactor designs focused only on components with measurable influence.

### Single-component variation identifies dominant contributors to medium performance

**Fig. 4:**
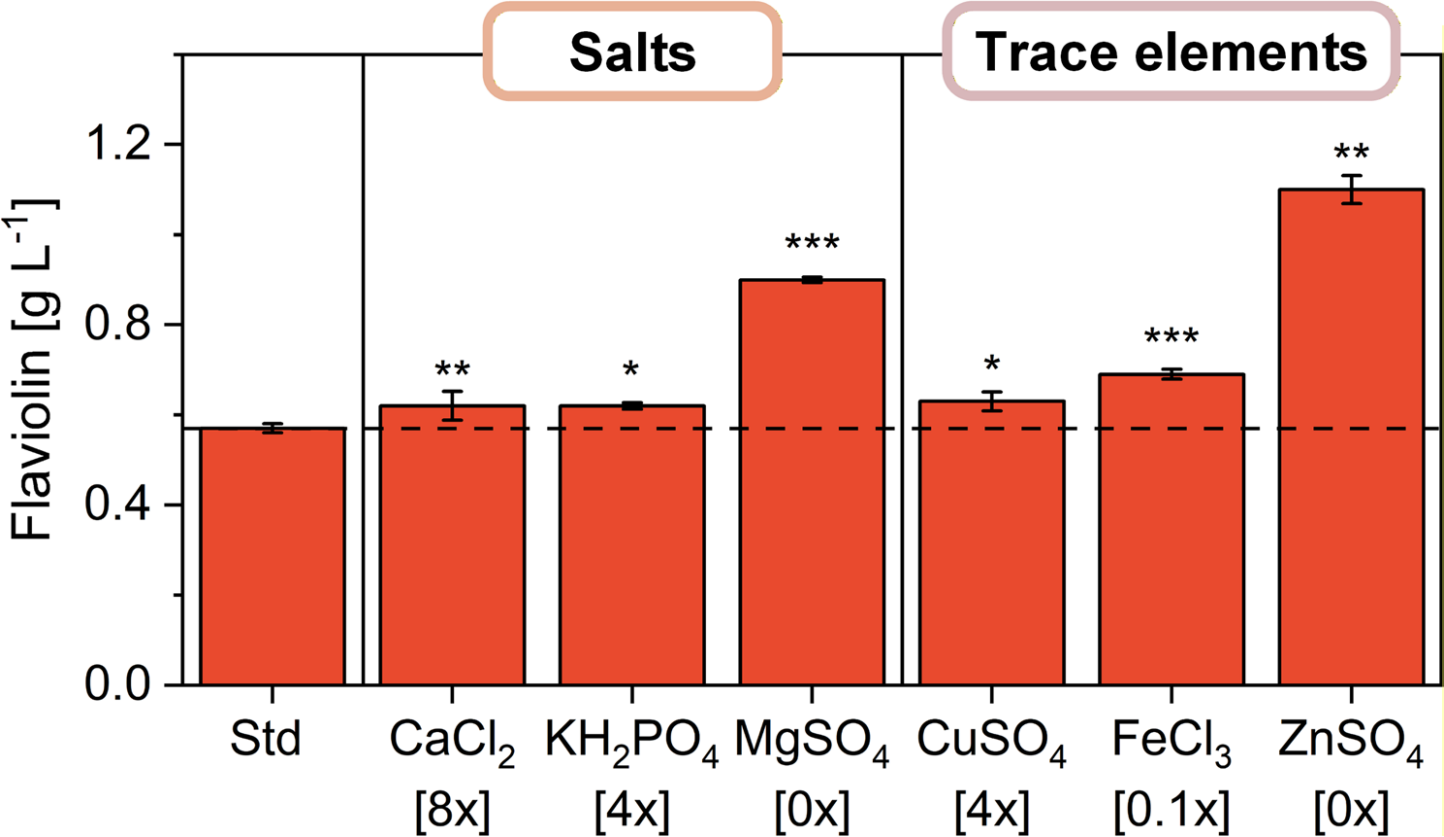
Modulation of YNB component concentrations using an OFAT approach. Maximum flaviolin titers achieved upon variation of individual salt and trace element levels, with the corresponding optimal concentrations shown in brackets. All cultivations were performed in micro-bioreactors in triplicates using a randomized design. Flaviolin titers were measured after 144 h. Statistical significance was assessed by two-sided *t*-tests comparing the indicated condition to the respective reference (1×) concentration and is denoted as *p* < 0.05 (*),* p* < 0.01 (**), and *p* < 0.001 (***). Detailed OFAT response curves for salts and trace elements are provided in the Supplementary Information (Additional File 1: Fig. S1 and Fig. S2)

To resolve the contribution of individual salts and trace elements, we next performed targeted single-component variation (OFAT) within the ranges identified in the group screen. This analysis revealed that, while most components had only minor or negligible effects - including NaCl and several trace elements such as Na_2_MoO_4_, NaI, H_3_BO_3_, and MnSO_4_, which all remained close to reference titers of 0.55 g L^− 1^ - a small subset strongly shaped flaviolin production (Fig. 4), with detailed OFAT response curves for salts and trace elements are shown in Fig. S1 and Fig. S2 (Additional File 1), respectively. Gradual reduction of ZnSO_4_ progressively increased titers, and complete omission yielded the highest levels of 1.10 g L^− 1^, identifying zinc as the dominant inhibitory factor under the tested conditions. Reduced MgSO_4_ similarly boosted production, reaching 0.90 g L^− 1^ in its absence, while elevating KH_2_PO_4_ increased titers to 0.70 g L^− 1^. In contrast, higher FeCl_3_ levels consistently decreased production, with 2× FeCl_3_ reducing titers to 0.40 g L^− 1^. These results defined the relevant concentration ranges, allowed several components to be deprioritized, and narrowed the factor space for subsequent multifactor designs, ensuring that the factorial and response surface analyses focused on components with measurable influence.

### Quantitative factorial design quantifies individual effects and ranks the most influential components

**Fig. 5:**
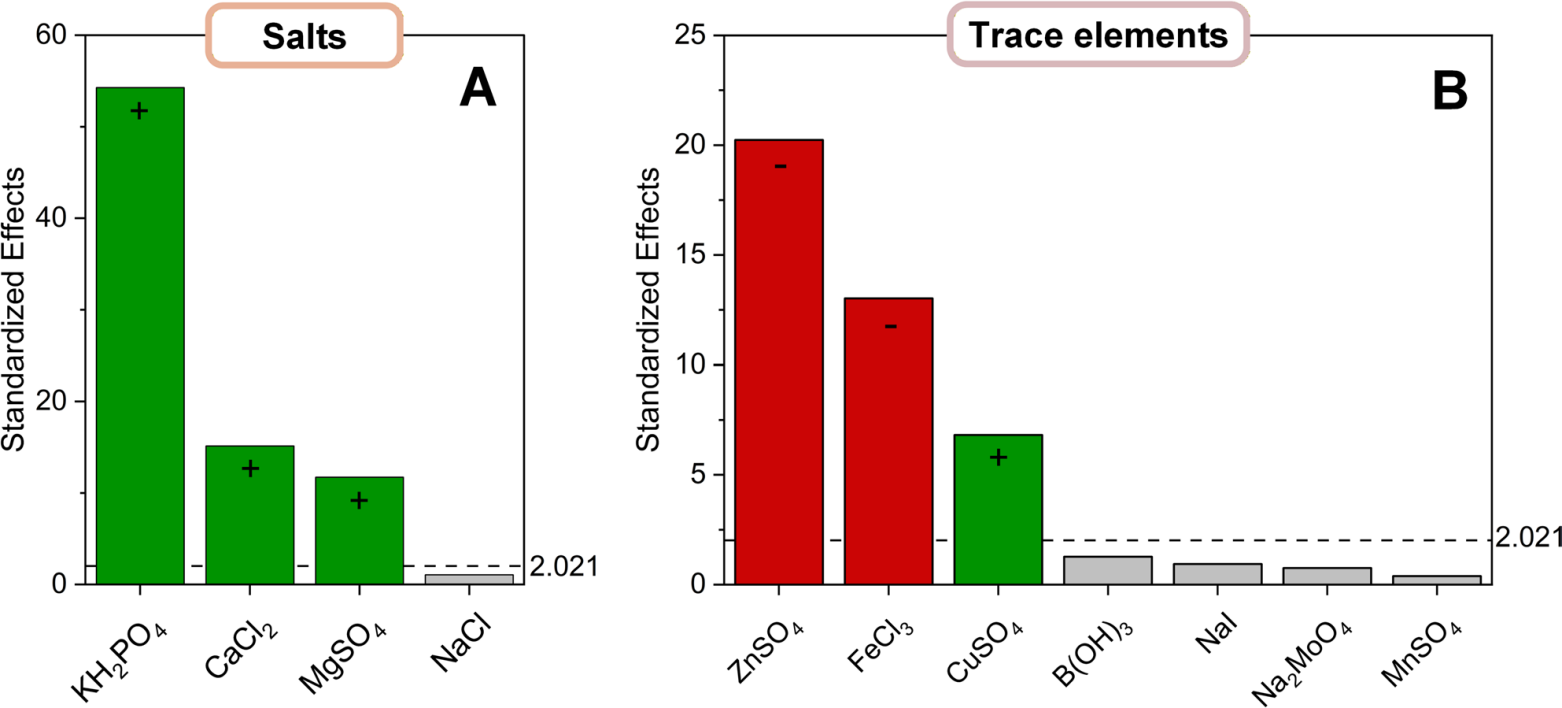
Factorial screening identifies the most influential salts and trace elements for flaviolin production. YNB salts and trace elements were evaluated in structured 2-level factorial designs. For the seven trace elements, a 1/8 fractional factorial design (Resolution IV; 16 base runs) with triplicates and three center points (51 total runs) was applied. For the four salts, a full factorial design (16 base runs) with triplicates and three center points (51 total runs) was used. Standardized effects on flaviolin titer are visualized as Pareto charts, with the significance threshold (α = 0.05) indicated. Green bars denote positive effects on flaviolin production, and red bars denote negative effects. Factors with bars extending beyond the significance line are considered statistically significant contributors to flaviolin formation within the tested ranges

Building on the OFAT results, the next step broadened the design space to determine whether combinations of components could further enhance performance. Multifactor interactions are common in mineral media, where ions affect uptake, enzyme activity, and cofactor availability in concert, meaning that single-factor tests may overlook synergistic or antagonistic effects. To systematically quantify these relationships and to rank the influence of individual factors, we applied a two-level Design of Experiments (DoE) approach. In total, 102 experimental runs were performed in this stage: a full factorial design for four salts (16 conditions + 1 center point, each in triplicate; 51 runs) and a 1/8 fractional factorial design for seven trace elements (16 conditions + 1 center point, each in triplicate; 51 runs). This structured design allowed us to resolve main effects reliably while efficiently probing potential interactions (Fig. 5).

The first-order linear models derived from these experiments describing flaviolin production under salt and trace element variation were:

1 $$ \begin{aligned} {\mathrm{Y}}_{{{\mathrm{Flaviolin}}}} [{\text{g L}}^{{ - {\kern 1pt} {\mathrm{1}}}} ]{\mkern 1mu} & = {\mkern 1mu} 0.{\mathrm{56}}{\mkern 1mu} + {\mkern 1mu} 0.0{\mathrm{8x}}_{{{\mathrm{KH2PO4}}}} \\ & + {\text{ }}0.0{\mathrm{2x}}_{{{\mathrm{MgSO4}}}} + {\text{ }}0.0{\mathrm{2x}}_{{{\mathrm{CaCl2}}}} + {\mkern 1mu} \varepsilon \\ \end{aligned} $$

2 $$ \begin{aligned} {\mathrm{Y}}_{{{\mathrm{Flaviolin}}}} [{\text{g L}}^{{ - {\kern 1pt} {\mathrm{1}}}} ]{\mkern 1mu} & = {\mkern 1mu} 0.{\mathrm{59}}{\mkern 1mu} + {\mkern 1mu} 0.0{\mathrm{3x}}_{{{\mathrm{CuSO4}}}} \\ & - 0.{\mathrm{1}}0{\mathrm{x}}_{{{\mathrm{ZnSO4}}}} - 0.0{\mathrm{6x}}_{{{\mathrm{FeCl3}}}} + \varepsilon \\ \end{aligned} $$

where x_i_ denotes the coded factor level and the coefficients represent the estimated linear contribution of each component. These equations formed the basis for calculating standardized effects (β-values), which were obtained by dividing each regression coefficient by its standard error. This standardization yielded dimensionless effect sizes that could be directly compared across factors with different concentration ranges and units. The resulting β-values quantified how strongly each component shifts flaviolin production within the tested design space and represented the natural starting point for constructing a steepest ascent path. For the steepest ascent method, the β-values were assembled into a direction vector corresponding to the gradient of maximum predicted increase in flaviolin titer. Step lengths for the individual components were then chosen proportional to the magnitude of their standardized effects, so that factors with large |β|-values (e.g., KH_2_PO_4_ and ZnSO_4_) were varied more strongly than weak contributors. This generated a series of medium formulations in which KH₂PO₄, MgSO_4_, CaCl_2_, CuSO_4_, ZnSO_4_, and FeCl_3_ were changed simultaneously but in β-scaled ratios, enabling an efficient traversal of the factor space toward improved performance.

Among the salts, KH_2_PO_4_ showed the strongest positive influence (β = + 0.42, *p* < 0.001), followed by smaller but still significant contributions from MgSO_4_ and CaCl_2_ (β = + 0.12 and + 0.10, *p* < 0.01). NaCl again showed no detectable effect (*p* = 0.46). For the trace elements, ZnSO_4_ emerged as the dominant inhibitory factor (β = − 0.55, *p* < 0.001), with FeCl_3_ also exerting a significant negative effect (β = − 0.33, *p* < 0.001). In contrast, CuSO_4_ displayed a modest stimulatory influence (β = + 0.18, *p* = 0.004). All remaining trace elements had b values below the significance threshold (*p* > 0.3), indicating negligible contributions within the tested range.

Importantly, factorial ANOVA revealed no major two-way interactions within the tested range (*p* > 0.1). This indicates that the strong performance shifts observed in the OFAT experiments were largely driven by individual components rather than synergistic effects between them. Together, these results confirmed the major drivers of medium performance and provided quantitative directionality for further optimization, enabling the subsequent use of model-guided strategies to approach an optimal medium formulation.

### Model-guided optimization narrows the search and reveals a nonlinear optimum

Using the quantitative effect estimates from the factorial design, we next applied a steepest ascent strategy to systematically move toward higher flaviolin production. In this approach, the concentrations of KH_2_PO_4_, MgSO_4_, CaCl_2_, CuSO_4_, ZnSO_4_, and FeCl_3_ were adjusted simultaneously and proportionally to their estimated influence, allowing the medium formulation to progress along the direction of predicted improvement. To define this direction quantitatively, the regression coefficients from the factorial models were first converted into standardized effects (β-values), which represent the slope of the response surface in coded units for each factor. These β-values constituted the gradient vector of the first-order model, and the principle of steepest ascent was to move proportionally along this gradient. This ensured that components with strong positive effects (e.g., KH_2_PO_4_) received larger upward adjustments, while components with strong negative effects (e.g., ZnSO_4_, FeCl_3_) were reduced more steeply.

**Fig. 6:**
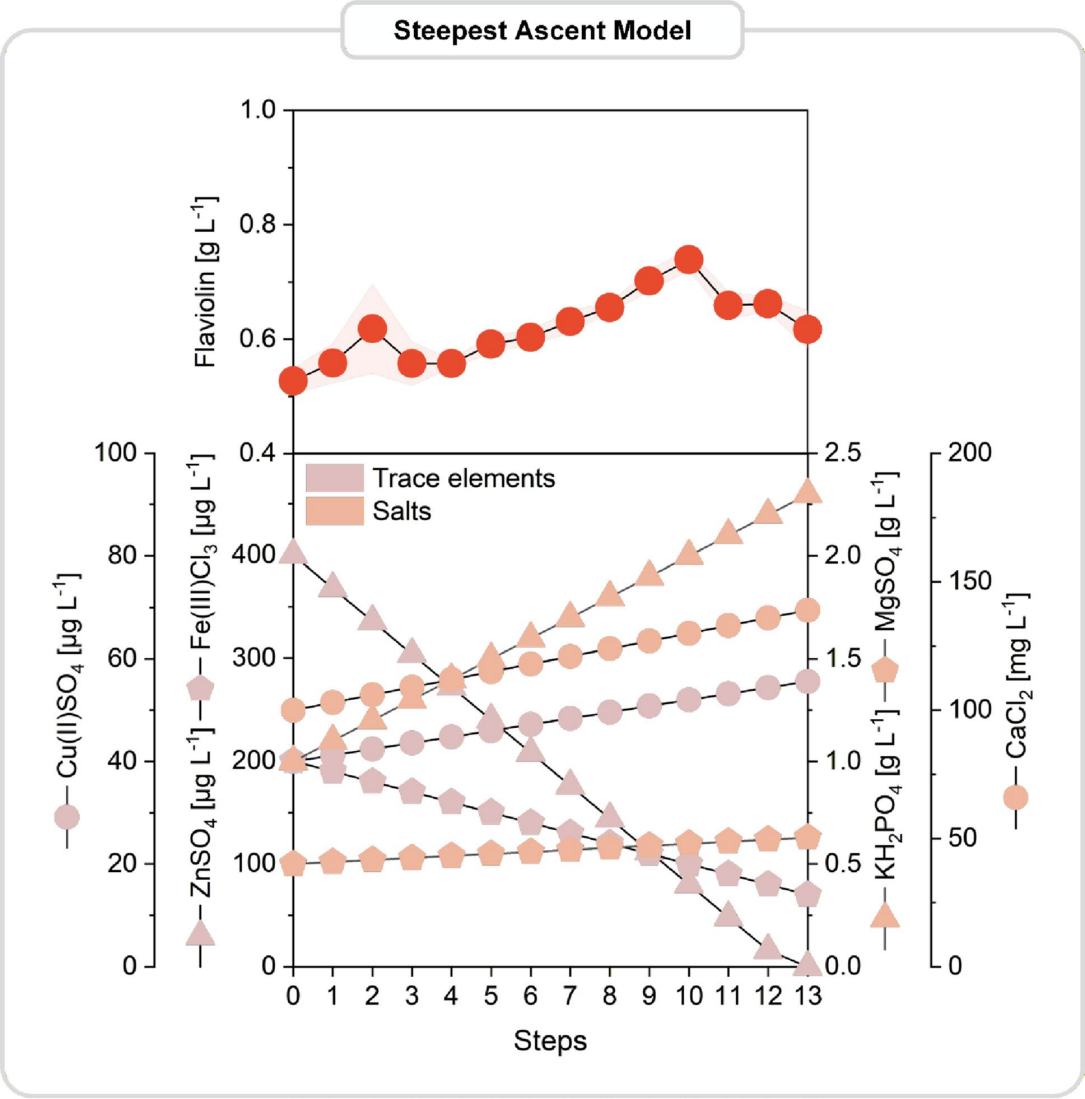
Steepest ascent optimization toward improved flaviolin production. Based on standardized effects estimated from the factorial designs, a steepest ascent method was used to move simultaneously along the direction of greatest predicted improvement by adjusting KH_2_PO_4_, MgSO_4_, CaCl_2_, CuSO_4_, ZnSO_4_, and FeCl_3_ concentrations. Fourteen consecutive medium formulations (steps 0–13) were generated by applying factor-specific step lengths, and each condition was cultivated in a micro-bioreactor in triplicate in randomized order. Flaviolin titers after 144 h are shown as means ± SD. The step with the highest flaviolin production defined the steepest ascent maximum (hill point) and served as the starting point for subsequent response surface modeling

Fourteen incremental medium formulations were tested in triplicate (42 new experiments in total), each representing a step along the calculated ascent path (Fig. 6). Along this guided trajectory, flaviolin production steadily increased and reached a maximum of 0.74 ± 0.02 g L^− 1^ at step 10 - a 1.4-fold improvement relative to the factorial starting point. Beyond this point, further adjustments reduced production, indicating that the ascent path had reached a local maximum within the linear region described by the factorial model. Importantly, this maximum remained below the 1.1 g L^− 1^ previously achieved by ZnSO_4_ omission alone, suggesting that the steepest ascent trajectory did not yet exploit the full improvement potential of the system. Such deviations between model-guided trajectories and empirical single-factor optima are common when underlying biological responses exhibit curvature or interaction effects that cannot be resolved by linear models [17, 18]. However, ZnSO_4_ omission alone did not identify the optimal concentrations of other essential minerals, and single-factor changes cannot establish whether combined adjustments unlock additional improvements or avoid hidden limitations. Together, these observations motivated a subsequent nonlinear exploration of the design space using response surface methodology.

To this end, we applied a Central Composite Design (CCD) to model nonlinear behavior and quantify both quadratic and interaction effects within the improved concentration range. The CCD consisted of 162 experimental runs in total, including a ½-fraction central composite layout with four blocks, triplicate measurements for all test conditions, eight center points per cube block, and two center points for the axial (star) block. The large number of runs ensured statistical robustness and reliable estimation of curvature, which is essential when optimizing multi-factor mineral media with potentially narrow optima, while the flaviolin reporter and the micro-bioreactor (microtiter plate–based) setup kept the experimental workload manageable, enabling the estimation of curvature and interaction effects that could not be detected in the linear models.

**Fig. 7:**
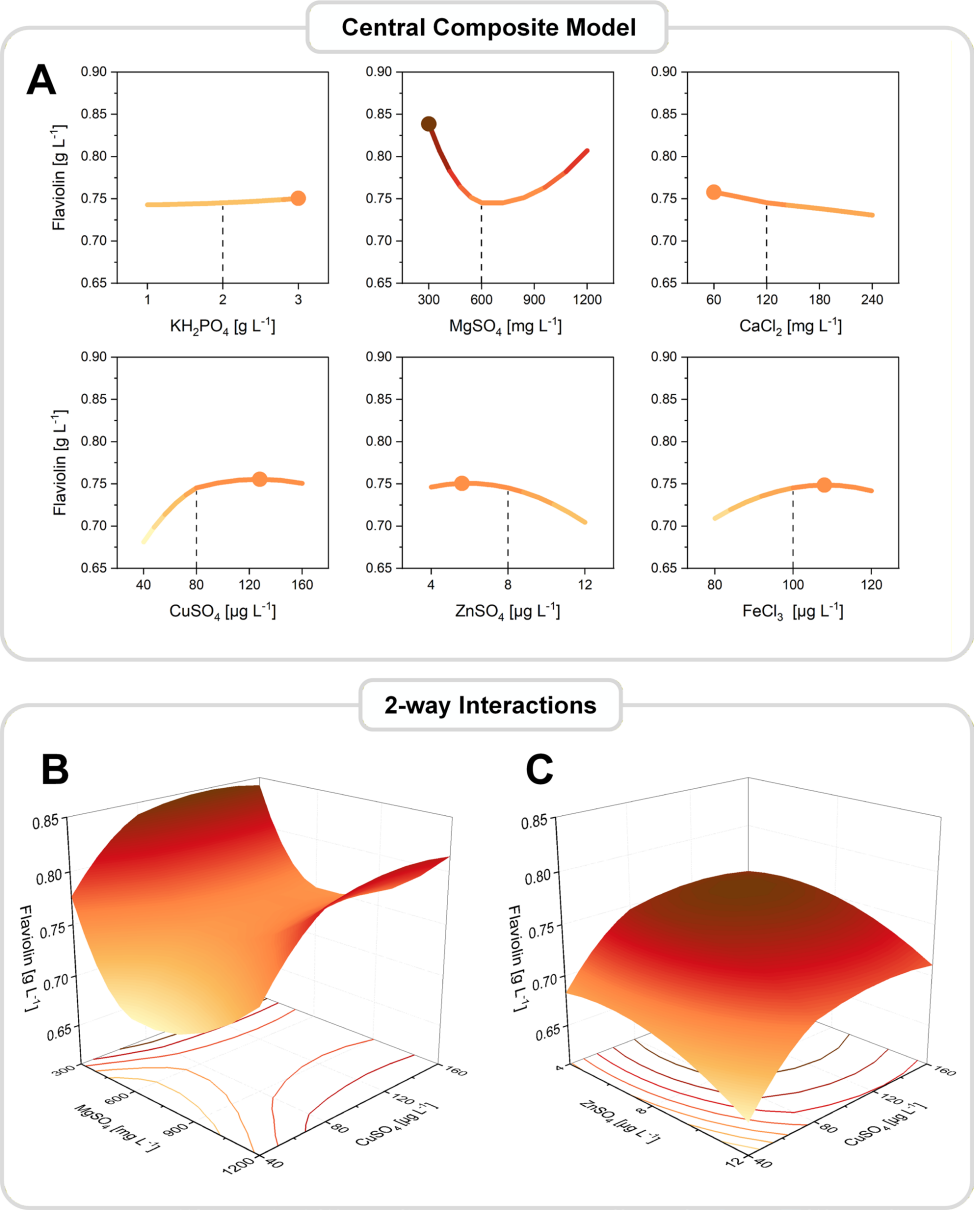
Response surface modeling of key mineral components and identification of a nonlinear optimum. A central composite design (CCD) was used to refine the concentration ranges of the six most influential components (KH_2_PO_4_, MgSO_4_, CaCl_2_, CuSO_4_, ZnSO_4_, FeCl_3_) around the steepest ascent maximum. The CCD (α = 1.5; 4 blocks; ½ fraction) comprised 162 randomized runs including factorial, axial (star), and center points, each in triplicate. Quadratic response surface models were fitted to flaviolin titers. Main-effect plots show the isolated influence of each component on flaviolin production, while two-factor response surfaces illustrate pairwise interactions and the location of the optimal region in the design space. In the main-effect plots, the dashed vertical line indicates the factor levels corresponding to the steepest ascent maximum (step 10), which was used as the center point for the CCD. The model-predicted optimum was used to formulate the final optimized synthetic mineral medium

The resulting response surface revealed clear optima for several components, resolving the bidirectional response of MgSO_4_, the inhibitory curvature of ZnSO_4_ and FeCl_3_, and the beneficial peak of CuSO_4_ (Fig. 7). Integrating these effects, the model predicted an optimal medium composition corresponding to a theoretical maximum of 1.04 ± 0.03 g L^− 1^, indicating that further improvements could be achieved by exploring nonlinear relationships beyond the steepest ascent trajectory. This value represents the model-derived stationary point of the response surface and does not correspond to a directly tested experimental condition but was obtained analytically from the fitted model parameters. Subsequent experimental validation near this predicted optimum yielded flaviolin titers exceeding the model prediction, consistent with the predicted optimum being located near the boundary of the explored design space.

### Experimental validation confirms model predictions and identifies a superior medium composition for flaviolin production

The CCD model not only predicted the presence of a nonlinear optimum but also provided explicit concentration targets for the six influential components. Based on the fitted response surface, the highest flaviolin production was predicted at markedly reduced levels of MgSO_4_, CaCl_2_, and ZnSO_4_, a moderate reduction of KH_2_PO_4_, and elevated concentrations of CuSO_4_ and FeCl_3_. In combination, these adjustments represented a medium composition substantially different from both the original YNB formulation and the steepest ascent trajectory, indicating a distinct optimal region in the design space.

**Fig. 8:**
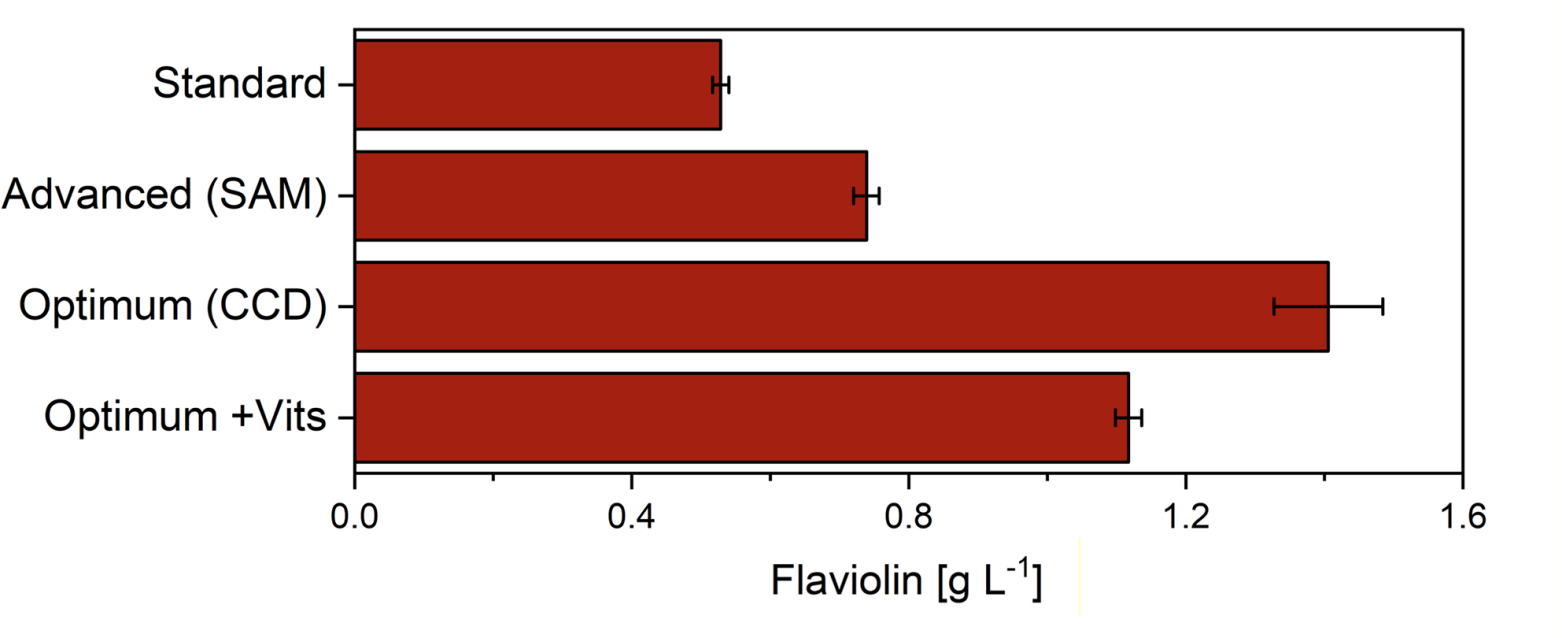
Validation of the optimized medium for flaviolin production. Flaviolin titers after 144 h micro-bioreactor cultivation. Production performance was compared under three medium conditions: (i) standard YNB component levels, (ii) the steepest ascent medium, (iii) the optimized mineral medium derived from the response surface model (RSM optimum), and (iv) the same optimized medium supplemented with a vitamin mixture corresponding to the vitamin content of the original YNB medium. Flaviolin titers were quantified after 144 h in micro-bioreactors. Data represent means ± SD of biological triplicates

To test these predictions experimentally, we formulated a medium according to the CCD-derived optimum and evaluated flaviolin production under the corresponding conditions (Fig. 8). Remarkably, the optimized formulation yielded 1.41 ± 0.08 g L^− 1^ flaviolin, exceeding the model’s theoretical maximum (1.04 g L^− 1^). This result not only validated the direction identified by the response surface model but also demonstrated that the model slightly underestimated the true peak performance - likely because several predicted optima were located at the boundaries of the experimental range, where model accuracy naturally decreases. Importantly, this optimized composition surpassed both the steepest ascent maximum (0.74 ± 0.02 g L^− 1^) and the best OFAT result obtained by omitting ZnSO_4_ (1.1 g L^− 1^), confirming that combined component adjustments unlock additional productivity gains that single-factor changes cannot achieve. Supplementing the optimized medium with a vitamin mixture did not improve production, confirming that vitamins are dispensable even under optimized mineral conditions. These findings highlight the value of an iterative, model-guided optimization strategy and establish the CCD-derived formulation as a robust candidate for a high-performance synthetic medium.

For the PIS-based hp16d–*rppA* strain, the optimized mineral medium also increased flaviolin production compared to the standard YNB formulation, although the relative fold-change was smaller than in hp16d–*rppA* C3-18 (Additional File 1: Fig. S3). This confirms that the identified mineral composition is beneficial for reporter strains with both defined (PIS) and random integration sites.

### Optimization strategy extends to LC-PUFA production in engineered *Y. lipolytica* strains

**Fig. 9:**
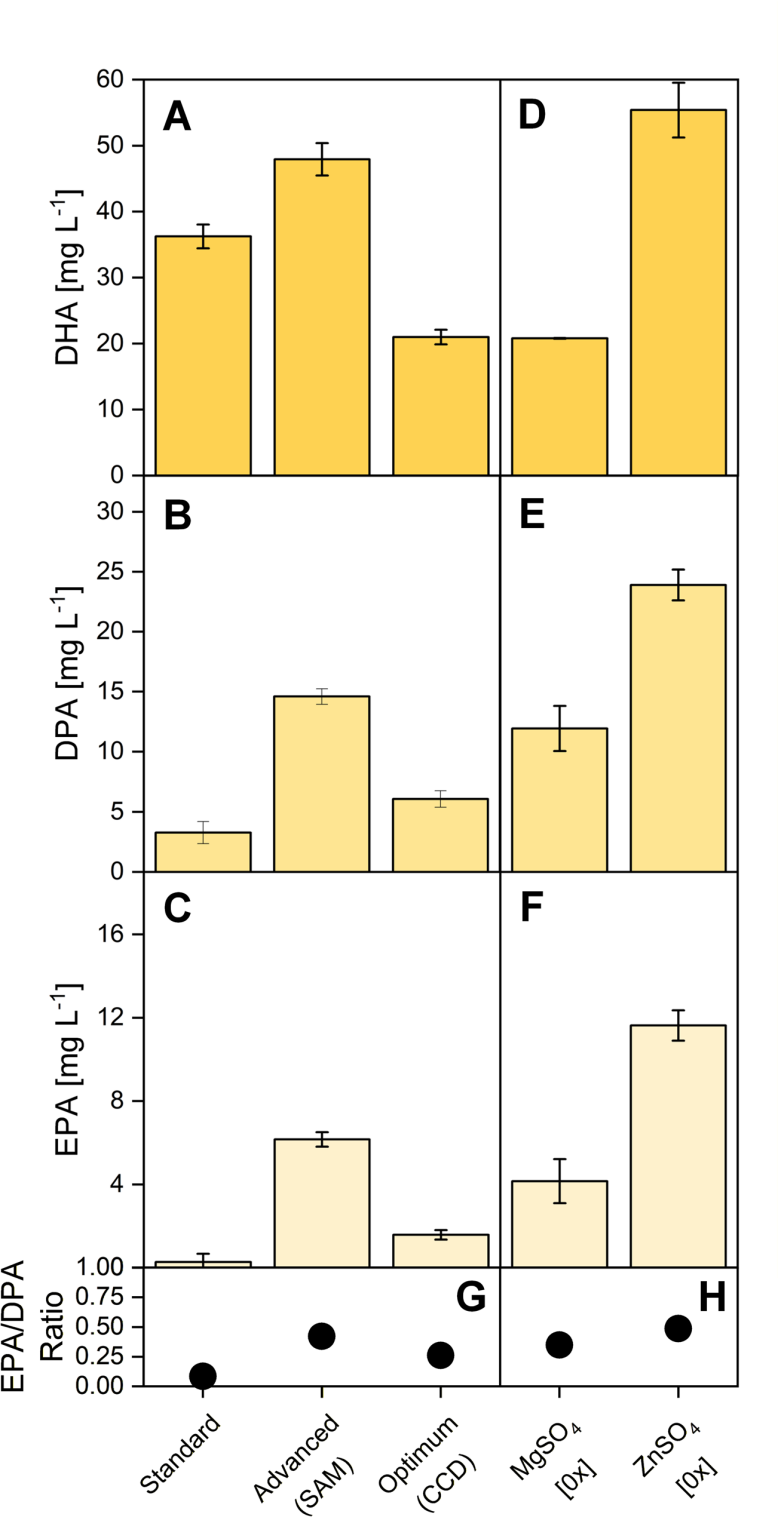
Impact of medium optimization on DHA, DPA, and EPA production in engineered *Y. lipolytica* strains. Titers of DHA, DPA, and EPA after 144 h micro-bioreactor cultivation for *Y. lipolytica* strains producing flaviolin and long-chain polyunsaturated fatty acids (LC-PUFAs): *Y. lipolytica rppA* (reporter), *Y. lipolytica* Af4 (DHA producer), and *Y. lipolytica* Ppt6 (DPA and EPA producer). Production performance was assessed under five mineral medium conditions: (i) standard YNB component levels, (ii) an intermediate steepest ascent condition, (iii) the maximized steepest ascent medium, (iv) the optimized synthetic mineral medium predicted by the response surface model (RSM optimum), and (v) a “no Zn” medium in which ZnSO_4_ was omitted while all other YNB components were maintained at standard levels. Data represent means ± SD of biological triplicates.* DHA* docosahexaenoic acid;* DPA* docosapentaenoic acid;* EPA* eicosapentaenoic acid;* LC-PUFA* long-chain polyunsaturated fatty acid

To assess whether the medium optimization developed for flaviolin production also benefits more complex acetyl-CoA/malonyl-CoA–derived pathways, we applied selected conditions to two LC-PUFA–producing *Y. lipolytica* strains: Af4 (DHA) and Ppt6 (EPA and DPA). Each strain was cultivated under standard mineral YNB (without vitamins), an intermediate and the maximum steepest ascent conditions, the CCD-derived theoretical optimum, and a “no Zn” condition in which ZnSO_4_ was omitted while all other YNB components remained at standard levels (Fig. 9).

LC-PUFA titers increased progressively from the standard formulation to the steepest ascent optimum, mirroring the trend observed for flaviolin and indicating that simultaneous adjustment of key salts and trace elements improves productivity across different product pathways (Fig. 9A–C). In contrast, applying the CCD theoretical optimum resulted in markedly reduced LC-PUFA synthesis. This condition drives MgSO_4,_ CaCl_2_, and ZnSO_4_ concentrations toward zero, and additional tests in which only MgSO_4_ was removed produced similarly low titers, identifying magnesium limitation as a major factor behind reduced LC-PUFA productivity (Additional File 1, Fig. S4).

The strongest improvements were obtained by omitting ZnSO_4_ while keeping all other mineral components constant (Fig. 9D–F). Under this condition, DHA increased from 36.6 to 56.7 mg L^− 1^ in strain Af4 (1.5-fold), while, in strain Ppt6 DPA increased from 3.1 to 23.6 mg L^− 1^ (7.6-fold), and EPA from 0.2 to 11.5 mg L^− 1^ (58-fold). These values represent the highest LC-PUFA titers observed in this study and highlight ZnSO_4_ omission as a simple and highly effective intervention to boost LC-PUFA production. Notably, the new medium also affected the DPA/EPA ratio (Fig. 9G, H).

## Discussion

### Mineral balancing as a key determinant of metabolic performance in *Y. lipolytica*

Our systematic deconstruction of YNB revealed that mineral composition is a major determinant of performance of acetyl-CoA– and malonyl-CoA–derived production pathways in engineered *Y. lipolytica*, shifting product formation by several-fold despite only modest changes in growth, effectively decoupling productivity from biomass formation. Medium composition is well known to exert profound control over physiological state and product yields in *Y. lipolytica* [8, 19], with environmental and nutritional parameters strongly shaping metabolic outputs and stress responses [20–22]. This aligns with reports that trace metal variability in minimal media markedly alters yeast physiology and undermines reproducibility [5, 11]. Such variability also has important implications for systems biology, as multi-omics datasets generated under nominally identical YNB conditions may differ substantially depending on trace metal composition, potentially confounding comparative analyses and biological interpretation [23, 24]. Consistent with earlier efforts to establish *Y. lipolytica* as a robust platform for lipid and PUFA production [9, 12, 25, 26], these findings identify mineral balancing as a powerful, actionable lever for process optimization.

At the same time, the present results underscore that optimal mineral compositions are inherently pathway-specific. Acetyl-CoA– and malonyl-CoA–derived pathways place particularly high demands on redox balance, metal-dependent enzymes, and cofactor regeneration, rendering them especially sensitive to zinc, iron, magnesium, and phosphate availability. Other product classes—such as amino acids, organic acids, or secreted enzymes—are likely to exhibit distinct mineral dependencies reflecting their specific enzymatic architectures and cellular bottlenecks. Consequently, a single universally optimal mineral medium is unlikely to exist even within a single host organism. Rather than proposing a one-size-fits-all solution, our work provides a structured and transferable workflow to identify pathway-tailored mineral compositions with high reproducibility. While alternative defined minimal media such as Delft medium [27] are also used for yeast cultivation, this study deliberately focused on YNB because it is the most widely applied defined mineral formulation in *Y. lipolytica*. The DoE-based workflow presented here is readily transferable to other defined media to identify pathway-specific mineral optima.

Among individual components, ZnSO_4_ and FeCl_3_ emerged as the strongest inhibitors, whereas KH_2_PO_4_ showed the strongest stimulatory effect. The inhibitory effect of zinc is well supported mechanistically: Zn^2+^ acts as a cofactor for numerous transcriptional regulators and metalloproteins and tightly interacts with fungal iron homeostasis [28]. Excess zinc suppresses high-affinity iron uptake and activates metal stress responses, disrupting Fe–S cluster assembly, mitochondrial respiration, and redox balance [28–30]. Because PKS and LC-PUFA pathways rely on iron-dependent desaturases and acyl-carrier-protein–linked reactions [9], removal of ZnSO_4_ likely alleviates metal stress and restores iron-dependent biosynthetic capacity, explaining the substantial increases in flaviolin and LC-PUFA production observed here.

Iron availability itself displayed a narrow functional window. While essential for Fe–S cluster biogenesis and electron transport, elevated FeCl_3_ consistently reduced flaviolin production, consistent with Fenton-driven reactive oxygen species formation that damages Fe–S proteins and impairs mitochondrial function [31]. As both polyketide and fatty acid synthesis depend on NADPH and reduced cofactors, oxidative stress is expected to suppress these pathways in favor of stress responses, explaining the inhibitory effect of excess iron. The CCD-derived optimum, which reduced but did not eliminate iron, supports the existence of a tight balance between deficiency and toxicity in *Y. lipolytica*, highlighting zinc and iron homeostasis as the dominant mineral determinants of pathway performance.

Beyond zinc and iron, magnesium emerged as a critical but pathway-dependent determinant. Mg^2+^ plays a central role in *Y. lipolytica* physiology as an essential cofactor for ATP-dependent enzymes, kinases, and polymerases, and for ribosome and nucleotide stabilization [32]. In acetyl-CoA– and malonyl-CoA-centered metabolism, magnesium is particularly important for sustaining high flux through glycolysis, acetyl-CoA formation, and malonyl-CoA synthesis. The observation that MgSO_4_ depletion improved flaviolin titers in the reporter-based CCD optimum but severely impaired LC-PUFA production highlights that reporter systems capture only a subset of pathway demands. Complex, multi-enzyme PUFA synthase systems place higher cumulative demands on energy metabolism and cofactor availability, likely making them more sensitive to magnesium limitation.

The correlated effects observed between Mg^2+^, Cu^2+^, and Zn^2+^ are likely driven by broader metal homeostasis and competition effects rather than direct pathway-specific interactions. Copper serves as a cofactor for mitochondrial enzymes and oxidative stress defense, while zinc excess perturbs iron and copper uptake and triggers metal stress responses [28, 30, 33]. Adjusting one metal therefore indirectly reshapes the intracellular availability and utilization of others, leading to coupled responses that become apparent only in multifactorial designs. These interactions illustrate why single-factor optimization is often insufficient for mineral media and underscore the value of structured DoE-based approaches.

Phosphate availability, modulated through KH_2_PO_4_, also exerted a strong positive effect on pathway performance. Phosphate is central to energy metabolism, nucleotide synthesis, buffering capacity, and redox cofactor cycling [34]. Increased KH_2_PO_4_ likely supports elevated acetyl-CoA and malonyl-CoA turnover by stabilizing cellular energy charge and intracellular pH, thereby enabling higher biosynthetic flux without proportionally increasing biomass formation. The observed plateauing response at higher phosphate levels suggests that phosphate acts as a permissive rather than strictly limiting factor once basic energetic demands are met.

Finally, the dispensability of vitamins observed here further illustrates the pathway specificity of medium requirements. Acetyl-CoA– and malonyl-CoA–derived pathways primarily rely on central carbon metabolism, redox balance, and metal-dependent enzymatic steps, and *Y. lipolytica* is capable of de novo synthesis of essential B-vitamins under standard laboratory conditions when cultivated on glycerol. Under these conditions, external vitamin supplementation is therefore not required to sustain precursor supply and may even impose indirect constraints, for example by affecting redox balance, metal availability, or regulatory signaling. In contrast, vitamin requirements are known to be critical for other applications, such as recombinant protein production, where high translational and secretory demands render vitamin supply limiting [20]. Similar dependencies are likely to arise under fast-growth regimes, nutrient limitation, or in auxotrophic backgrounds. These differences emphasize that vitamin and mineral requirements cannot be generalized across production targets but must be evaluated in the context of pathway architecture, cellular burden, and cultivation conditions.

### A simplified synthetic medium improves reproducibility, cost efficiency, and process robustness

Beyond increasing product titers, the optimized mineral formulation also resolved a major practical limitation of standard YNB: batch-to-batch variability. Commercial YNB mixtures differ markedly in trace metal content across suppliers and lots, leading to inconsistent growth and metabolic performance [5]. This variability directly translated into 1.5–2-fold differences in DHA production and growth in our comparative experiments, complicating process development and quantitative strain evaluation. By reconstructing YNB from defined individual components, we eliminated this source of variation and achieved highly consistent performance across independently prepared batches.

An additional advantage of the simplified formulation is its markedly reduced cost. Commercial YNB represents one of the most expensive components in defined yeast media due to bundled vitamins and complex trace element mixes. In contrast, the optimized formulation omits all vitamins and relies only on inexpensive commodity-grade salts and trace metals added to standard carbon, nitrogen, and sulfur sources. A simple cost comparison indicates that mineral-related medium costs can be reduced by an order of magnitude relative to standard YNB, with no negative impact on growth and substantial gains in product formation. This aligns with industrial medium development principles, where cost efficiency, raw material availability, and component control represent key drivers alongside process performance [11]. These considerations become even more relevant during scale-up, particularly in bioprocesses that utilize process water, agricultural streams, or technical-grade substrates, which frequently contain variable amounts of iron, zinc, and other metal ions. Such background contamination has been shown to inhibit yeast fermentation and lipid production in industrial processes, particularly when Fe and Zn levels fluctuate [35, 36]. Monitoring metal content and adjusting supplementation or employing standard industrial mitigation strategies such as controlled mineral feeds or water treatment will help maintain performance during scale-up.

Finally, the defined composition facilitates regulatory compliance [11]. Component-level control simplifies raw material qualification, supplier auditing, and documentation of lot consistency—key requirements in regulated industrial and food- and pharma-related fermentations [37]. Eliminating complex and biologically derived vitamin mixtures reduces variability and simplifies traceability, supporting medium standardization and technology transfer. Together, the reduced cost, improved reproducibility, and compatibility with industrial raw materials position the simplified medium as a practical and transferable option for larger-scale *Y. lipolytica* bioprocesses.

### Pathway-specific optimization reveals broader applicability to complex biosynthetic systems

The medium optimization itself was conducted with a high-output reporter strain (hp16d–*rppA* C3-18) carrying the *rppA* cassette at a randomly integrated locus, chosen to maximize signal-to-noise and dynamic range in the DoE workflow. Nonetheless, the optimized formulation and its key features also improved flaviolin production in the original PIS-based hp16d-*rppA* reporter. This indicates that the identified mineral effects are robust across different integration contexts and are not an artefact of a single genomic insertion event.

The benefits of the optimized mineral formulation extended beyond the flaviolin reporter system and translated to strains producing long-chain polyunsaturated fatty acids (LC-PUFAs). These pathways represent some of the most demanding metabolic modules implemented in *Y. lipolytica*, relying on large, multi-domain megasynthases and tightly regulated, cofactor-dependent reactions [10, 12]. The pronounced improvements in DHA, DPA, and EPA production therefore provide a stringent validation that the optimized medium enhances not only simple polyketide synthesis but also complex, multi-enzyme biosynthetic systems.

Interestingly, the medium conditions that maximized flaviolin production did not fully coincide with those supporting the highest LC-PUFA titers. Similar observations have been reported for PUFA-producing *Y. lipolytica* strains, where medium composition strongly affected product formation [12, 15]. This divergence underscores that distinct acetyl-CoA– and malonyl-CoA–derived pathways respond differently to mineral composition, likely reflecting differences in enzyme architecture, cofactor requirements, and metabolic integration. Consequently, a universal medium optimum across products is unlikely, even within a single host background. Rather than seeking a single medium formulation suitable for all pathways, our results demonstrate that the structured workflow developed here can rapidly identify pathway-specific optima. The successfully transferred optimized medium - and simple, high-impact interventions such as ZnSO_4_ omission - to multiple PUFA-producing strains illustrates the general applicability of this approach and highlights medium engineering as a powerful complement to genetic optimization.

Importantly, reliance on YNB-based defined media is not limited to flaviolin or PUFA production (Table 1). Standard YNB formulations are widely used as production media in *Y. lipolytica* for lipids, hydrocarbons, carotenoids, and polyketides [6, 7, 38], and they remain among the dominant defined media in other industrial yeasts including *Saccharomyces cerevisiae* [39], *Komagataella pastoris* [40], and *Kluyveromyces marxianus* [41]. The finding that a single mineral intervention—ZnSO_4_ omission—increased DHA and DPA titers up to 7.6-fold and EPA titers up to 58-fold highlights the potential of targeted mineral balancing to unlock latent production capacity even in complex pathways.

Given the widespread use of YNB-based media across yeasts and process stages, the medium design principles established here are likely transferable to a broad range of microbial cell factories and offer an actionable starting point for systematic medium optimization in both academic and industrial settings. Together, these findings demonstrate that tailored mineral balancing can substantially elevate production in both simple and complex biosynthetic pathways in *Y. lipolytica*, and that model-guided medium engineering represents a broadly applicable tool for improving performance across diverse metabolic architectures.

## Conclusions

This study establishes a systematic and highly effective strategy for optimizing mineral media for acetyl-CoA– and malonyl-CoA–derived production in *Y. lipolytica*. By dissecting the contribution of each salt and trace element and progressively refining their concentrations through model-guided design, we developed a simplified synthetic medium that eliminates unnecessary vitamins and strategically tunes mineral levels. Hereby, the sequential DoE strategy was not redundant but hierarchical: OFAT reduced complexity, linear models and steepest ascent provided directionality, and CCD resolved nonlinear optima once the relevant design space had been identified. This rational optimization approach increased flaviolin titers by more than threefold and, importantly, proved transferable to complex biosynthetic pathways such as long-chain polyunsaturated fatty acid synthesis. Because the formulation is constructed from chemical commodity chemicals, it can be directly prepared in any laboratory without dependency on expensive proprietary YNB premixes. The finding that a single targeted intervention - ZnSO_4_ omission - substantially improved LC-PUFA production highlights the versatility and practical applicability of the strategy across different metabolic architectures and strain backgrounds. The optimized synthetic mineral medium provides a robust and reproducible baseline for bioprocess development, offering a practical starting point that balances performance gains with ease of implementation.

Overall, this work underscores the value of component-resolved medium engineering and demonstrates its potential to unlock latent production capabilities in *Y. lipolytica*. The presented workflow provides a blueprint for designing tailored media for diverse acetyl-CoA– and malonyl-CoA–dependent products paving the way toward more efficient, reproducible, and economically viable bioprocesses based on *Y. lipolytica* and other non-conventional yeast cell factories.

## Materials and methods

### Microorganisms, plasmids, and synthetic genes

*Escherichia coli* DH10B (Thermo Fisher Scientific, Waltham, MA, USA) was used for cloning. The basic *Y. lipolytica* strain Po1h (CLIB 882), the DHA producer *Y. lipolytica* Af4, and the DPA/EPA producer *Y. lipolytica* Ppt6 were taken from previous work [9]. Strains were maintained as glycerol stocks at − 80 °C. The plasmids pClik5a MCS [42], pACYC_assembly, and pKG2-PIS, allowing for site-specific integration into locus YALI0_C05907g [9], were used for gene assembly. The *rppA* gene (SGR_RS33090) from *Streptomyces griseus* subsp. *griseus* NBRC 13,350 was synthesized (ATG: biosynthetics GmbH, Merzhausen, Germany). All strains used in this study are listed in the Supplement (Additional File 1, Table S4).

### Growth media and mineral medium preparation

*Y. lipolytica* Po1h was grown in complex YPD medium containing 10 g L^− 1^ yeast extract (Becton Dickinson, Heidelberg, Germany), 20 g L^− 1^ peptone (Becton Dickinson), and 20 g L^− 1^ glucose. All other *Y. lipolytica* strains were cultivated in a chemically defined medium containing 1.7 g L^− 1^ YNB (either self-made (DIY) or a commercial premix; Becton Dickinson, MP Biomedicals, Irvine, CA, USA, or Sigma-Aldrich, Darmstadt, Germany), 10 g L^− 1^ glycerol, 5 g L^− 1^ (NH_4_)_2_SO_4_, and 200 mM MES (pH 6.7). For solid media, 20 g L^− 1^ agar (Becton Dickinson) was added. For plasmid selection in *E. coli*, 50 µg mL^− 1^ ampicillin, 25 µg mL^− 1^ chloramphenicol, or 50 µg mL^− 1^ kanamycin were used, as appropriate.

For all medium-optimization experiments, a defined mineral base (self-made YNB) was prepared from 20 individual component stock solutions. Stock solutions were prepared at 100× concentration for trace elements, vitamins and salts, using analytical-grade chemicals and deionized water (18.2 MΩ·cm). Individual components were dissolved separately to avoid precipitation, sterilized by filtration (0.22 μm), aliquoted, and stored at 4 °C. Immediately before use, the required volumes of stock solutions were combined to obtain the desired final concentrations. When the optimized synthetic medium was used, specific components (e.g. ZnSO_4_) were omitted or adjusted according to the experimental design.

For comparison experiments, commercial YNB premixes (Becton Dickinson; MP Biomedicals, Irvine, CA, USA; Sigma-Aldrich, Darmstadt, Germany) were dissolved directly in deionized water at 1.7 g L^− 1^ and sterilized by filtration (0.22 μm). No additional trace elements or vitamins were added. The use of component-resolved stock solutions ensured precise and reproducible control over mineral composition, eliminated batch-to-batch variation inherent to commercial YNB powders, and allowed accurate reconstruction of optimized formulations. Full formulations, component concentrations, and stock solution recipes are provided in the supplement (Additional File 1: Table S1, Table S2).

### Molecular design and genetic engineering

Cloning strategies were designed using SnapGene 7 (Insightful Science, San Diego, CA, USA). The assembly workflow for the *rppA* expression cassette was as follows. The minLEU2–*rppA*–Lip2t fragment was excised from the synthesized vector and inserted into pClik5a MCS by restriction/ligation using PacI and SmaI. Subsequently, UAS1B4, UAS1B8, or UAS1B16 elements were inserted into the SmaI site by Gibson assembly, yielding the expression cassettes hp4d–*rppA*–Lip2t, hp8d–*rppA*–Lip2t, and hp16d–*rppA*–Lip2t. These cassettes were excised with AvrII and PacI and ligated into pACYC_assembly (AvrII/PacI), and the resulting constructs were then cloned into pKG2-PIS using SdaI and PacI. PCR and Sanger sequencing (Genewiz, Leipzig, Germany) were used to verify each construct. Plasmids were transformed into *E. coli* DH10B by heat shock for amplification and isolation.

### Gene cassette integration into *Y. lipolytica* and strain selection

Transformation of *Y. lipolytica* was performed according to a previously described protocol [43] with minor adaptations. Briefly, fresh competent cells (5 × 10⁷) were resuspended in 600 µL lithium acetate solution (0.1 M, pH 6.0) and incubated for 1 h at 28 °C. Cells were collected (4000 × g, 1 min, room temperature) and resuspended in 40 µL LiAc solution. To this suspension, 10 µL herring testes carrier DNA (10 mg mL^− 1^ in TE buffer, denatured) and 500 ng linearized DNA were added, followed by incubation for 15 min at 28 °C. Then, 350 µL LiAc–PEG solution (40% PEG 4000 in 0.1 M lithium acetate, pH 6.0) were added and the mixture was incubated for 1 h at 28 °C. After addition of 40 µL DMSO, cells were heat shocked at 39 °C for 10 min, resuspended in 600 µL LiAc solution, and plated on YNB plates. After 3–4 days, colonies were screened by colony PCR for correct integration into the PIS site.

To obtain a producer strain with higher *rppA* expression for medium optimization, the hp16d–*rppA* cassette was also introduced without flanking homology regions to promote random chromosomal integration via non-homologous end joining (NHEJ). Transformation was performed using the same lithium acetate/PEG protocol described above, except that the linear cassette was used without PIS-targeting arms. Transformants were plated on YNB selection medium and evaluated for flaviolin production by colony color intensity, resulting in the selection of strain C3-18 as the high-production reporter strain for all subsequent medium-optimization experiments. Genomic PCR confirmed cassette integration and strain stability over multiple passages. All selected strains were further characterized in shake flasks starting from an initial OD_600_ of 0.1.

### Cultivation in the micro-bioreactor system

Micro-bioreactor cultivations were performed in 48-well plates (Biolector I, Beckman Coulter GmbH, Baesweiler, Germany) at 28 °C, 85% relative humidity, and 1200 rpm [44]. Cultures were grown for six days. Precultures were inoculated from single colonies on 2-day plate cultures, grown overnight, harvested (4000 × g, 1 min, room temperature), and used to inoculate the main cultures to a starting OD_600_ of 0.1. All conditions were conducted in biological triplicates.

### Cultivation in shake flasks

Shake flask experiments were conducted in 500 mL baffled flasks containing 50 mL medium on an orbital shaker (5 cm shaking diameter, 230 rpm; Multitron, Infors AG, Bottmingen, Switzerland) at 28 °C and 80% relative humidity. Precultures were inoculated from single colonies on 2-day plate cultures, grown overnight, harvested (4000 × g, 1 min, room temperature), and used to inoculate the main cultures to a starting OD_600_ of 0.1. All cultivations were performed in biological triplicates.

### Determination of the cell concentration

Cell concentration was determined from optical density measurements at 600 nm. An experimentally established correlation for *Y. lipolytica* was used to calculate cell dry weight (CDW) from OD_600_ readings: CDW [g L^− 1^] = 0.424 × OD_600_ [45].

### Quantification of flaviolin

Flaviolin concentrations in culture supernatants were quantified by measuring absorbance at 520 nm and applying the published extinction coefficient (1305 L mol^− 1^ cm^− 1^) [46].

### Quantification of glycerol

Glycerol was quantified by HPLC (Agilent 1200 series, Agilent Technologies, Waldbronn, Germany) using an anion exchange column (Aminex HPX-87 H, 300 × 7.8 mm, Bio-Rad, Hercules, CA, USA) at 45 °C with 12 mM H₂SO₄ as mobile phase at 0.5 mL min^− 1^. Analytes were detected by refractive index and quantified using external standards.

### Extraction and transesterification of fatty acids

For fatty acid analysis, 5 mg CDW were transferred into a glass vial, collected (12 000 × g, 5 min, 4 °C), and dried in a vacuum concentrator (Savant DNA 120 SpeedVac, Thermo Fisher) for 60 min at 65 °C and 9 mbar. Then, 300 µL of a methanol: toluene:95% sulfuric acid mixture (50:50:2, v/v/v) were added for simultaneous extraction and transesterification to fatty acid methyl esters (FAMEs). n-3 heneicosapentaenoic acid methyl ester (HPA, 21:5; Cayman Chemical, Ann Arbor, MI, USA) was added as internal standard. Samples were incubated at 80 °C for 24 h. After cooling to room temperature, reactions were neutralized with 250 µL stopping solution (0.5 M NH_4_HCO_3_ and 2 M KCl in H₂O). Following phase separation (12 000 × g, 5 min, room temperature), the organic phase was collected for GC-MS analysis.

### Analysis of fames by GC-MS

The analysis was conducted on a GC-MS instrument (6890 GC, 5973 inert MSD, Agilent Technologies) as described previously [12, 15].

### Design of experiments (DoE)

Medium optimization was performed using a structured Design of Experiments (DoE) workflow [47]. In this work, it consisted of component-group screening, one-factor-at-a-time (OFAT) variation, factorial screening, steepest ascent, and response surface modeling. First, a full factorial design (3³, 27 conditions) evaluated the effects of vitamins, salts, and trace elements at 0.5x, 1x, and 2x of standard concentrations, revealing salts and trace elements as dominant factors. Subsequently, individual components within these groups were varied using an OFAT approach, in which each salt or trace element was adjusted between 0x and up to 8x while all others remained constant. Components showing significant effects (*p* < 0.05) were selected for multifactor analysis. To quantify main effects and detect potential interactions, a two-level full factorial design (2⁴ + center points, 51 runs) was applied to four salts, and a two-level fractional factorial design (Resolution IV, 1/8 fraction, 51 runs) was applied to seven trace elements. The entire statistical dataset of the full factorial design is provided in Additional File 2.

### Calculation of regression coefficients and determination of the steepest-ascent direction

For both factorial designs, first-order regression models were fitted in coded units:

3 $$\:\mathrm{Y}={\mathrm{b}}_{0}+\sum\:_{\mathrm{i}}{\mathrm{b}}_{\mathrm{i}}{\mathrm{x}}_{\mathrm{i}},$$

where Y is the flaviolin titer, x_i_ the coded factor levels (− 1, 0, + 1), and b_i_ the estimated main effects. Coefficients were obtained by least-squares estimation, and significance was assessed by ANOVA (α = 0.05). The standardized coefficients (b_i_) served as quantitative measures of factor influence within the linear design space. The direction for the steepest ascent method (SAM) was calculated by scaling each factor in proportion to the square root of the sum of all effects:

4 $$\:{\Delta\:}{\mathrm{x}}_{\mathrm{i}}=\frac{{\lambda\:b}_{i}}{\sqrt{{\sum\:}_{j=1}^{k}{b}_{j}^{2}}}\:$$

with l chosen to yield experimentally feasible step sizes (l = 0.1) and k is the total number of factors. Positive coefficients led to increased factor levels and negative coefficients to decreased levels. These increments were used to generate 14 consecutive medium formulations evaluated in Fig. 6, progressively approaching regions of higher flaviolin production.

### Response surface modeling and identification of the predicted optimum

To identify nonlinear optima beyond the linear region explored by SAM, a central composite design (CCD) with six continuous factors (− 1.5 to + 1.5 coded levels) was conducted, comprising 162 randomized runs including factorial, axial, and center points. A quadratic response surface model (RSM) was fitted:

5 $$\:\mathrm{Y}={\mathrm{b}}_{0}+\sum\:_{\mathrm{i}}{\mathrm{b}}_{\mathrm{i}}{\mathrm{x}}_{\mathrm{i}}+\sum\:_{\mathrm{i}}{\mathrm{b}}_{\mathrm{i}\mathrm{i}}{\mathrm{x}}_{\mathrm{i}}^{2}+\sum\:_{\mathrm{i}<\mathrm{j}}{\mathrm{b}}_{\mathrm{i}\mathrm{j}}{\mathrm{x}}_{\mathrm{i}}{\mathrm{x}}_{\mathrm{j}}$$

Curvature and interactions were evaluated by ANOVA and model diagnostics. The stationary point of the fitted surface was obtained by solving

6 $$\:\nabla\:\mathrm{Y}=0$$

and converted back to actual concentrations, yielding the RSM-predicted optimum that was subsequently validated experimentally (Fig. 8). All DoE designs and statistical analyses were generated using OriginPro 2024 (Design of Experiments App, OriginLab Corporation). Details of the experimental designs are provided in the supplement (Additional file 1, Table S3).

## Supplementary Information

Below is the link to the electronic supplementary material.

Supplementary Material 1.

Supplementary Material 2.

## Data Availability

All data generated or analyzed during this study are included in this published article and its supplementary information.
