# Supplementary material for: Defined YNB-free mineral medium improves reproducibility and enables high-titer production in Yarrowia lipolytica
Source: Microb Cell Fact. 2026 Feb 3;25:59. doi: 10.1186/s12934-026-02939-6 (PMC12930956; doi:10.1186/s12934-026-02939-6)
Supplement: Supplementary file 1 — Supplementary Material 1. [file 12934_2026_2939_MOESM1_ESM.pdf]

Additional File 1 to:

Defined YNB-free mineral medium improves reproducibility and enables high-titer production in *Yarrowia lipolytica*

Demian Dietrich, Hang Qi, Sofija Jovanovic Gasovic, Michael Kohlstedt, and Christoph Wittmann#

Institute of Systems Biotechnology, Saarland University, Saarbrücken, Germany

Contact information

demian.dietrich@gmail.com

hang.qi@uni-saarland.de

sofijajg@uic.edu

michael.kohlstedt@uni-saarland.de

christoph.wittmann@uni-saarland.de

Phone/Fax: +49 681 302 71970 / 71972

Table S1. Stock solutions used for preparation of DIY (Do-it-yourself) YNB and optimized synthetic mineral media. Recipes and handling instructions for all stock solutions used in this study. Individual compound stocks were prepared at 100x and combined to yield the final media compositions listed in Table S2. Standard concentrations (1x) were referenced from Sigma-Aldrich (Prod. No. Y1251). The minimum amount weighed for each compound was 5 mg to ensure sufficient accuracy. Reported weights refer to the anhydrous chemical formula; they must be adjusted accordingly when using salts with water of crystallization (e.g. hydrates). 1) Store at 4 °C and in the dark. 2) Sterilize by filtration. 3) Sterilize by autoclaving.

Vitamins	Medium conc.	Stock conc.	Preparation	Comments
Calcium pantothenate	400 µg/L	40 mg/L (100x)	20 mg, ad. 500 mL H2O	1), 2)
Inositol	2000 µg/L	200 mg/L (100x)	100 mg, ad. 500 mL H2O	1), 2)
Niacin	400 µg/L	40 mg/L (100x)	20 mg, ad. 500 mL H2O	1), 2)
p-Aminobenzoic acid	200 µg/L	20 mg/L (100x)	10 mg, ad. 500 mL H2O	1), 2)
Pyridoxine hydrochloride	400 µg/L	40 mg/L (100x)	20 mg, ad. 500 mL H2O	1), 2)
Riboflavin	200 µg/L	20 mg/L (100x)	10 mg, ad. 500 mL H2O	1), 2)
Thiamine hydrochloride	400 µg/L	40 mg/L (100x)	20 mg, ad. 500 mL H2O	1), 2)
Biotin	2 µg/L	0.2 mg/L (100x)	20 mg, ad. 1000 mL H2O (10000x). Dilute 1:100	1), 2)
Folic acid	2 µg/L	0.2 mg/L (100x)	20 mg, ad. 1000 mL H2O (10000x). Dilute 1:100	1), 2), Adjust to pH=9
**Trace elements**				
Boric acid	500 µg/L	50 mg/L (100x)	50 mg, ad. 1000 mL H2O	1), 2)
Copper sulfate	40 µg/L	4 mg/L (100x)	8 mg, ad. 2000 mL H2O	1), 2)
Natrium Iodide	100 µg/L	10 mg/L (100x)	10 mg, ad. 1000 mL H2O	1), 2)
Ferric chloride	200 µg/L	20 mg/L (100x)	10 mg, ad. 500 mL H2O	1), 2), Adjust to pH=1
Manganese sulfate	400 µg/L	40 mg/L (100x)	40 mg, ad. 1000 mL H2O	1), 2)
Sodium molybdate	200 µg/L	20 mg/L (100x)	20 mg, ad. 1000 mL H2O	1), 2)
Zinc sulfate	400 µg/L	40 mg/L (100x)	40 mg, ad. 1000 mL H2O	1), 2)
**Salts**				
Potassium phosphate monobasic	1 g/L	100 g/L (100x)	10 g, ad. 100 mL H2O	1), 3)
Magnesium sulfate	0.5 g/L	50 g/L (100x)	5 g, ad. 100 mL H2O	1), 3)
Sodium chloride	0.1 g/L	10 g/L (100x)	1 g, ad. 100 mL H2O	1), 3)
Calcium chloride	0.1 g/L	10 g/L (100x)	1 g, ad. 100 mL H2O	1), 3)

Table S2. Composition of defined mineral media used in this study. Overview of all YNB-based and YNB-free mineral media employed in flaviolin and LC-PUFA production experiments. Media are grouped into (i) commercial YNB-based formulations, (ii) lab-made component-resolved YNB (DIY YNB) and variants, and (iii) optimized synthetic mineral media. The presence/absence of vitamins and ZnO_4 is listed for each formulation. All media contained 10 g L^{-1} glycerol as carbon source, 5 g L^{-1} $(NH_4)_2SO_4$ as nitrogen source and were buffered at pH 6.7 (200 mM MES). Detailed stock solution recipes are given in Table S1, and factor ranges used in Design of Experiments (DoE) are summarized in Table S3.

ID	Medium name	YNB source / type	$ZnSO_4$	Vitamins	Notes / where used
M1	Commercial YNB1 + glycerol	YNB1 (Manufacturer 1, Lot1)	Yes	Yes	Batch variability (Fig. 1)
M2	Commercial YNB2 + glycerol	YNB2 (Manufacturer 1, Lot2)	Yes	Yes	Batch variability (Fig. 1)
M3	Commercial YNB3 + glycerol	YNB3 (Manufacturer 1, Lot3)	Yes	Yes	Batch variability (Fig. 1)
M4	Commercial YNB4 + glycerol	YNB4 (Manufacturer 2, Lot1)	Yes	Yes	Batch variability (Fig. 1)
M5	Commercial YNB5 + glycerol	YNB5 (Manufacturer 2, Lot2)	Yes	Yes	Batch variability (Fig. 1)
M6	Commercial YNB6 + glycerol	YNB6 (Manufacturer 3, Lot1)	Yes	Yes	Batch variability (Fig. 1)
M7	DIY YNB (standard)	Component-resolved (DIY)	Yes	Yes	Batch variability (Fig. 1), Group-wise screening 1x condition (Fig. 3)
M8	Salt/trace OFAT medium (standard reference)	Component-resolved (DIY)	1×	No	Reference for Fig. 4, S1, S2
M9	Steepest ascent – intermediate step	Component-resolved (DIY)	Varied	No	Condition in Fig. 6, Fig. 9
M10	Steepest ascent – maximum (step 10)	Component-resolved (DIY)	Varied	No	Hill point in Fig. 6, used in Fig. 8, Fig. 9
M11	CCD theoretical optimum	CCD-derived mixture	Near 0	No	CCD optimum, used in Fig. 9
M12	RSM-optimized mineral medium (final flaviolin medium)	RSM optimum mixture	Near 0	No	Main optimized medium (Fig. 8, 9)
M13	RSM optimum + vitamins	RSM optimum + vitamin mix	Near 0	Yes	Vitamin test on optimized medium (Fig. 8)
M14	“No Zn” medium	DIY YNB w/o $ZnSO_4$	No	No	Strongest LC-PUFA improvement (Fig. 9)
M15	MgSO4 omission medium	DIY YNB w/o $MgSO_4$	Yes	No	Compared to CCD optimum (Fig. S4)

Table S3. Overview of DoE designs. Summary of design type, purpose, factors, levels, and run structure for each DoE stage.

Design ID	DoE stage	Design type (notation)	Factors included	Levels per factor	Total conditions (base runs)	Center points	Replicates	Total runs
D1	Group screen	Full factorial 3^3	Vitamins, salts, trace elements at group level	0.5×, 1×, 2×	27	0	2	54
D2	OFAT salts	One-factor-at-a-time	KH2PO4, MgSO4, CaCl2, NaCl	0–8× (stepwise)	24	0	3	72
D3	OFAT trace elements	One-factor-at-a-time	CuSO4, FeCl3, ZnSO4, Na2MoO4, NaI, B(OH)3, MnSO4	0–8× (stepwise)	33	0	3	99
D4	Salt factorial	Full factorial 2^4 + center points	KH2PO4, MgSO4, CaCl2, NaCl	−1, +1, 0 (center)	16	3	3	51
D5	Trace element factorial	Fractional factorial 2^{7-4} (Res. IV)	CuSO4, FeCl3, ZnSO4, Na2MoO4, NaI, B(OH)3, MnSO4	−1, +1, 0 (center)	16	3	3	51
D6	Steepest ascent	Path of steepest ascent (linear model)	KH2PO4, MgSO4, CaCl2, CuSO4, FeCl3, ZnSO4	14 steps along gradient	14	0	3	42
D7	CCD / RSM	½-fraction Central Composite Design	KH2PO4, MgSO4, CaCl2, CuSO4, FeCl3, ZnSO4	−1.5, −1, 0, +1, +1.5	32 + 12 [cube + star]	8 + 2 [cube + star]	3	162

Table S4. Strains used in this study

Strain	Genotype	Role	Origin
E. coli			
DH10B	F- mcrA Δ(mrr-hsdRMS-mcrBC) φ80lacZΔM15	Cloning host	Thermo Fisher
Y. lipolytica			
Po1h	eu2-270 ura3-302 xpr2-322	Base strain	[1]
hp4d–rppA	Po1h, PIS::hp4d–rppA–Lip2t; Leu^{+}	Construct evaluation	This study
hp8d–rppA	Po1h, PIS::hp8d–rppA–Lip2t; Leu^{+}	Construct evaluation	This study
hp16d–rppA	Po1h, PIS::hp16d–rppA–Lip2t; Leu^{+}	Construct evaluation	This study
C3-18	Po1h, random integration of hp16d–rppA–Lip2t	Flaviolin reporter	This study
Af4	Po1h, PUFA synthase cassette for DHA	DHA producer	[2]
Ppt6	Po1h, PUFA synthase cassette for EPA/DPA	EPA/DPA producer	[1]

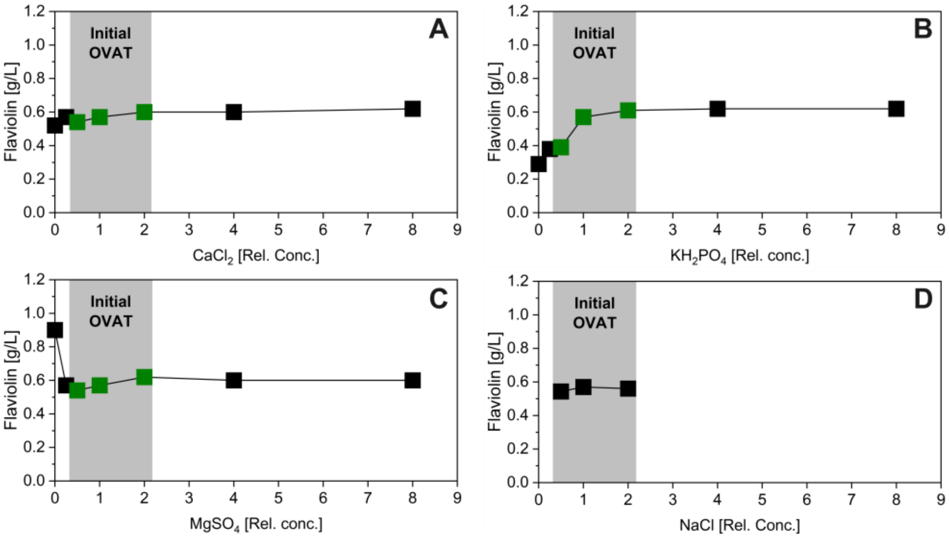

Figure S1. OFAT variation of YNB salts reveals strong differential effects on flaviolin production. The concentration of each salt was varied individually while all other YNB components were maintained at their standard level (1×). Initial screening tested 0.5×, 1×, and 2× concentrations (grey area). Data points are color-coded according to their correlation with flaviolin production (green, positive; red, negative). Significance was assessed by two-sided t-tests comparing 0.5× and 2× concentrations ($\alpha = 0.05$). KH_2PO_4 showed a significant positive effect ($p = 1.2 \times 10^{-3}$), increasing flaviolin up to 25% relative to 1×. $MgSO_4$ showed a highly significant inhibitory effect ($p = 4.6 \times 10^{-6}$), with complete omission increasing titers up to 0.90 ± 0.01 g L^{-1} (1.6-fold vs. reference). $CaCl_2$ showed no significant effect ($p = 0.08$). NaCl showed no measurable influence ($p = 0.46$). When significant differences were detected, additional concentrations were evaluated to refine the response curves and identify optimal ranges. Cultivations were performed in micro-bioreactors for 144 h in randomized order. Data represent means ± SD of biological triplicates (n = 3). No correction for multiple testing was applied because concentrations and hypotheses were predefined based on group-level screening, and OFAT results served exclusively to reduce factor space for subsequent factorial and response surface designs.

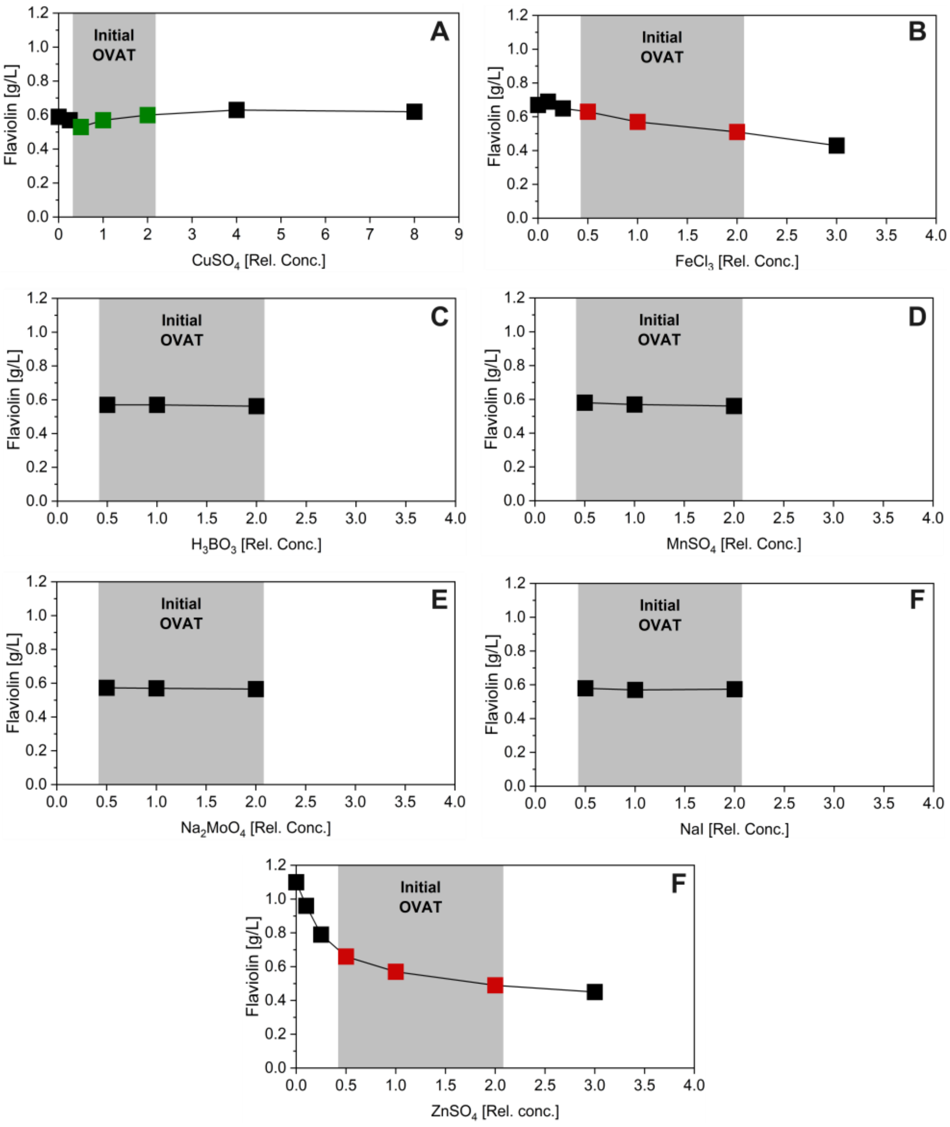

Figure S2. OFAT variation of YNB trace elements identifies zinc and iron as dominant inhibitory factors. Trace element concentrations were varied individually while all other components were maintained at standard levels (1×). Initial screening tested 0.5×, 1×, and 2× concentrations (grey area). Data points are color-coded according to their correlation with flaviolin production (green, positive; red, negative). Significance was assessed by two-sided t-tests comparing 0.5× and 2× concentrations ($\alpha = 0.05$). $CuSO_4$ showed a mild but significant stimulatory effect ($p = 0.03$), increasing titers by 10%. $FeCl_3$ displayed a strong inhibitory trend ($p = 3.1 \times 10^{-5}$), with 2× $FeCl_3$ reducing titers to 0.40 g L^{-1} (30% decrease). $ZnSO_4$ showed the strongest inhibitory response ($p = 9.4 \times 10^{-8}$), with complete omission yielding the highest titers across all OFAT tests (1.10 ± 0.02 g L^{-1}; 2-fold vs. reference). Na_2MoO_4, NaI, $B(OH)_3$, and $MnSO_4$ exhibited no significant effects (all $p > 0.1$). When significant differences were detected, additional concentrations were evaluated to determine optimal ranges. Cultivations were performed in micro-bioreactors for 144 h in randomized order. Data represent means ± SD of biological triplicates ($n = 3$). No correction for multiple testing was applied because concentrations and hypotheses were predefined based on group-level screening, and OFAT results served exclusively to reduce factor space for subsequent factorial and response surface designs.

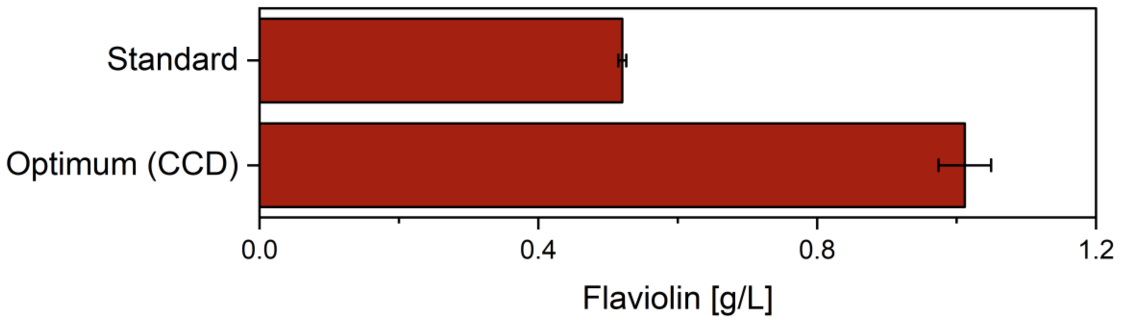

Figure S3. Effect of the optimized mineral medium on flaviolin production in the PIS-based hp16d–rppA strain. The PIS-based reporter strain (Po1h, PIS::hp16d–*rppA*–Lip2t) was cultivated in micro-bioreactors for 144 h in standard DIY YNB-based mineral medium (with vitamins) and in the optimized synthetic mineral medium (RSM optimum). Flaviolin titers were quantified in the supernatant at 520 nm. The optimized medium increased flaviolin production relative to the standard formulation, confirming that key mineral trends identified with the high-output reporter strain C3-18 also apply to a defined single-copy PIS integration. Data represent means ± SD of biological triplicates (n = 3).

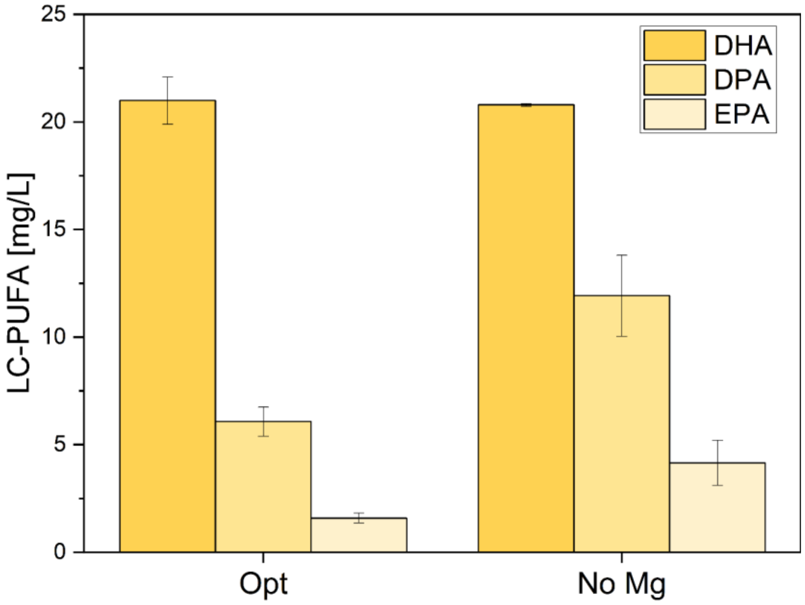

Figure S4. Effect of $MgSO_4$ omission on PUFA production compared to the CCD-derived theoretical optimum. Long-chain PUFA production was compared between the CCD-predicted optimum medium and a medium in which $MgSO_4$ was omitted while all other mineral components remained at standard levels. DHA, DPA, and EPA titers were quantified after 144 h in micro-bioreactors for strains Af4 (DHA) and Ppt6 (EPA/DPA). Omission of $MgSO_4$ markedly reduced PUFA synthesis in both strains (DHA: −45%, $p = 2.8 \times 10^{-4}$; EPA: −92%, $p = 6.1 \times 10^{-6}$; DPA: −78%, $p = 1.3 \times 10^{-5}$), identifying magnesium limitation as a major contributor to the poor performance observed under the CCD theoretical optimum. Cultivations were performed in randomized order in micro-bioreactors. Data represent means ± SD of biological triplicates (n = 3).

References

1. Gemperlein K, Dietrich D, Kohlstedt M, Zipf G, Bernauer HS, Wittmann C, Wenzel SC, Muller R: **Polyunsaturated fatty acid production by Yarrowia lipolytica employing designed myxobacterial PUFA synthases.** *Nat Commun* 2019, **10:**4055.
2. Dietrich D, Jovanovic Gasovic S, Cao P, Kohlstedt M, Wittmann C: **Refactoring the architecture of a polyketide gene cluster enhances docosahexaenoic acid production in *Yarrowia lipolytica* through improved expression and genetic stability.** *Microb Cell Fact* 2023, **22:**199.
