# Supplementary material for: Defined YNB-free mineral medium improves reproducibility and enables high-titer production in Yarrowia lipolytica
Source: Microb Cell Fact. 2026 Feb 3;25:59. doi: 10.1186/s12934-026-02939-6 (PMC12930956; doi:10.1186/s12934-026-02939-6)
Supplement: Supplementary file 2 — Supplementary Material 2. [file 12934_2026_2939_MOESM2_ESM.pdf]

# Additional File 2 to:

# Defined YNB-free mineral medium improves reproducibility and enables high-titer production in *Yarrowia lipolytica*

Demian Dietrich, Hang Qi, Sofija Jovanovic Gasovic, Michael Kohlstedt, and Christoph Wittmann#

Institute of Systems Biotechnology, Saarland University, Saarbrücken, Germany

Contact information

demian.dietrich@gmail.com

hang.qi@uni-saarland.de

sofijajg@uic.edu

michael.kohlstedt@uni-saarland.de

christoph.wittmann@uni-saarland.de

# Phone/Fax: +49 681 302 71970 / 71972

**Table S1. Experimental design of the full factorial screening for YNB compound classes.** Overview of the full factorial Design of Experiments (DoE) used to evaluate the effects of YNB compound classes on flaviolin production. Three factors—vitamins, trace elements, and salts—were investigated at three concentration levels (0.5×, 1×, and 2× relative to standard YNB). The design comprised 27 unique factor combinations, each tested in duplicate, resulting in 54 total experimental runs. The execution order was fully randomized to minimize systematic bias and time-dependent effects.

| **Design** | Full Factorial |
|---|---|
| **Factors** | 3 |
| **Number of Levels** | 3,3,3 |
| **Base Runs** | 27 |
| **Total Runs** | 54 |
| **Replicates** | 2 |
| **Randomize Design** | Yes |

**Table S2. Experimental matrix and flaviolin titers from the full factorial YNB compound class screening.** Complete experimental matrix of the $3^3$ full factorial design showing the relative concentration levels of vitamins, trace elements, and salts tested in each run. Each condition was cultivated in biological duplicate. Flaviolin titers (g $L^{-1}$) were determined after 144 h of micro-bioreactor cultivation. Each row represents an individual experimental condition; variability and significance across the design space were assessed by factorial ANOVA rather than replicate-based standard deviations.

| Replicates | Vitamins | Trace elements | Salts | Flaviolin [g/L] |
|---|---|---|---|---|
| 1 | 0.5 | 0.5 | 0.5 | 0.45 |
| 1 | 0.5 | 0.5 | 1 | 0.53 |
| 1 | 0.5 | 0.5 | 2 | 0.71 |
| 1 | 0.5 | 1 | 0.5 | 0.29 |
| 1 | 0.5 | 1 | 1 | 0.45 |
| 1 | 0.5 | 1 | 2 | 0.62 |
| 1 | 0.5 | 2 | 0.5 | 0.13 |
| 1 | 0.5 | 2 | 1 | 0.32 |
| 1 | 0.5 | 2 | 2 | 0.46 |
| 1 | 1 | 0.5 | 0.5 | 0.45 |
| 1 | 1 | 0.5 | 1 | 0.52 |
| 1 | 1 | 0.5 | 2 | 0.70 |
| 1 | 1 | 1 | 0.5 | 0.29 |
| 1 | 1 | 1 | 1 | 0.45 |
| 1 | 1 | 1 | 2 | 0.60 |
| 1 | 1 | 2 | 0.5 | 0.13 |
| 1 | 1 | 2 | 1 | 0.31 |
| 1 | 1 | 2 | 2 | 0.43 |
| 1 | 2 | 0.5 | 0.5 | 0.45 |
| 1 | 2 | 0.5 | 1 | 0.53 |
| 1 | 2 | 0.5 | 2 | 0.66 |
| 1 | 2 | 1 | 0.5 | 0.30 |
| 1 | 2 | 1 | 1 | 0.43 |
| 1 | 2 | 1 | 2 | 0.58 |
| 1 | 2 | 2 | 0.5 | 0.13 |
| 1 | 2 | 2 | 1 | 0.31 |
| 1 | 2 | 2 | 2 | 0.40 |
| 2 | 0.5 | 0.5 | 0.5 | 0.48 |
| 2 | 0.5 | 0.5 | 1 | 0.54 |
| 2 | 0.5 | 0.5 | 2 | 0.72 |
| 2 | 0.5 | 1 | 1 | 0.47 |
| 2 | 0.5 | 1 | 2 | 0.59 |
| 2 | 0.5 | 2 | 0.5 | 0.16 |
| 2 | 0.5 | 2 | 1 | 0.30 |
| 2 | 1 | 0.5 | 0.5 | 0.48 |
| 2 | 1 | 0.5 | 2 | 0.74 |
| 2 | 1 | 1 | 1 | 0.44 |
| 2 | 1 | 2 | 0.5 | 0.13 |
| 2 | 1 | 2 | 1 | 0.31 |
| 2 | 1 | 2 | 2 | 0.47 |
| 2 | 2 | 0.5 | 0.5 | 0.51 |
| 2 | 2 | 0.5 | 1 | 0.56 |
| 2 | 2 | 0.5 | 2 | 0.71 |
| 2 | 2 | 1 | 0.5 | 0.30 |
| 2 | 2 | 1 | 1 | 0.45 |
| 2 | 2 | 2 | 0.5 | 0.13 |
| 2 | 2 | 2 | 1 | 0.31 |
| 2 | 2 | 2 | 2 | 0.47 |

**Table S3. Statistical quality metrics of the full factorial model for YNB compound class screening.** Statistical summary of the linear model fitted to the full factorial experimental data. Reported parameters include degrees of freedom, root mean square error (RMSE), coefficient of determination ($R^2$), adjusted $R^2$, and residual sum of squares. The high $R^2$ and low RMSE indicate an excellent model fit and high explanatory power of the selected factors within the explored design space.

| | |
|---|---|
| **Degrees of Freedom** | 21 |
| **Root Mean Square of Error** | 0.02214 |
| **R-Square** | 0.99229 |
| **Adj. R-Square** | 0.98275 |
| **Residual Sums of Squares** | 0.01029 |

**Table S4. Analysis of variance (ANOVA) for the full factorial YNB compound class screening.** Results of the factorial ANOVA evaluating the effects of vitamins, trace elements (TE), and salts on flaviolin production. Degrees of freedom (DF), sum of squares, mean square, F-values, and p-values are reported for main effects, two-way interactions, and the three-way interaction. Statistically significant effects ($p < 0.05$) demonstrate that trace elements and salts are the dominant determinants of flaviolin production, whereas vitamins exert only a minor influence within the tested concentration range.

| | DF | Sum of Squares | Mean Square | f-value | p-value |
|---|---|---|---|---|---|
| **Vitamins** | 2 | 0.00595 | 0.00297 | 6.06696 | 0.00833 |
| **TE** | 2 | 0.68677 | 0.34338 | 700.6545 | 5.98227E-20 |
| **Salts** | 2 | 0.60818 | 0.30409 | 620.4742 | 2.10071E-19 |
| **Vitamins x TE** | 4 | 3.56124E-4 | 8.90311E-5 | 0.18166 | 0.94534 |
| **Vitamins x Salts** | 4 | 0.00166 | 4.13876E-4 | 0.84449 | 0.51271 |
| **TE x Salts** | 4 | 0.02066 | 0.00516 | 10.53815 | 7.63384E-5 |
| **Vitamins x TE x Salts** | 8 | 0.00113 | 1.40876E-4 | 0.28745 | 0.96264 |
| **Error** | 21 | 0.01029 | 4.9009E-4 | | |
| **Total** | 47 | 1.33498 | | | |
